# Neuroanatomical changes observed over the course of a human pregnancy

**DOI:** 10.1038/s41593-024-01741-0

**Published:** 2024-09-16

**Authors:** Laura Pritschet, Caitlin M. Taylor, Daniela Cossio, Joshua Faskowitz, Tyler Santander, Daniel A. Handwerker, Hannah Grotzinger, Evan Layher, Elizabeth R. Chrastil, Emily G. Jacobs

**Affiliations:** 1 Department of Psychological & Brain Sciences, University of California, Santa Barbara, CA USA; 2 Department of Neurobiology and Behavior, University of California, Irvine, CA USA; 3 Section on Functional Imaging Methods, Laboratory of Brain and Cognition, National Institute of Mental Health, National Institutes of Health, Bethesda, MD USA; 4 Neuroscience Research Institute, University of California, Santa Barbara, CA USA

**Keywords:** Neuroscience, Brain

## Abstract

Pregnancy is a period of profound hormonal and physiological changes experienced by millions of women annually, yet the neural changes unfolding in the maternal brain throughout gestation are not well studied in humans. Leveraging precision imaging, we mapped neuroanatomical changes in an individual from preconception through 2 years postpartum. Pronounced decreases in gray matter volume and cortical thickness were evident across the brain, standing in contrast to increases in white matter microstructural integrity, ventricle volume and cerebrospinal fluid, with few regions untouched by the transition to motherhood. This dataset serves as a comprehensive map of the human brain across gestation, providing an open-access resource for the brain imaging community to further explore and understand the maternal brain.

## Abstract (web summary)

Neural changes in pregnancy are not well understood. Here Pritschet et al. present an open-access precision brain imaging resource, mapping neuroanatomical change in an individual from preconception through postpartum.

## Main

Worldwide, nearly 85% of women experience one or more pregnancies in their lifetime^1^, with 140 million women becoming pregnant each year. Over an approximately 40-week gestational window, the maternal body undergoes profound physiological adaptations to support the development of the fetus, including increases in plasma volume, metabolic rate, oxygen consumption and immune regulation^2^. These rapid adaptations are initiated by 100-fold to 1,000-fold increases in hormone production, including estrogen and progesterone. These neuromodulatory hormones also drive significant reorganization of the central nervous system. Evidence from animal models and human studies converge on pregnancy as a period of remarkable neuroplasticity^3–10^ (see ref. ^10^ for one of the earliest known observations). Gestational increases in steroid hormone synthesis drive neurogenesis, dendritic spine growth, microglial proliferation, myelination and astrocyte remodeling (for review, see ref. ^11^). These cellular changes are pronounced in brain circuits that promote maternal behavior. For example, Ammari et al. recently discovered that steroid hormones can fine-tune the response properties of galanin neurons in the rodent medial preoptic area of the hypothalamus (mPOA), leading to enhanced sensitivity in dams to sensory cues from newborn pups^12^.

In humans, reductions in gray matter volume (GMV) have been observed postpartum^13–16^, particularly in regions central to theory-of-mind processing^13^. These GMV changes persist at 6 years postpartum^17^ and are traceable decades later^18,19^, underscoring the permanence of this major remodeling event. And yet the changes that occur within the maternal brain during gestation itself are virtually unknown (see ref. ^20^ for early neuroimaging insight). A recent study by Paternina-Die et al. offers intriguing clues^21^. Women were scanned once in the third trimester and again in the postpartum period, revealing a reduction of cortical volume observable in the late pregnancy scan. These findings suggest that pregnancy is a highly dynamic period for neural remodeling, yet neuroscientists lack a detailed map of how the human brain changes throughout the gestational period.

Here we conducted a precision imaging study of pregnancy in which a healthy 38-year-old primiparous woman underwent 26 magnetic resonance imaging (MRI) scans and venipuncture beginning 3 weeks preconception through 2 years postpartum. We observed widespread reductions in cortical GMV and cortical thickness (CT) occurring in step with advancing gestational week and the dramatic rise in sex hormone production. Remodeling was also evident within subcortical structures, including the ventral diencephalon, caudate, thalamus, putamen and hippocampus. High-resolution imaging and segmentation of the medial temporal lobe (MTL) extend these findings further, revealing specific volumetric reductions within hippocampal subfields CA1, CA2/CA3 and parahippocampal cortex (PHC). In contrast to widespread decreases in cortical and subcortical GMV, correlational tractography analyses revealed nonlinear increases in white matter quantitative anisotropy (QA) throughout the brain—indicating greater tract integrity—as gestational week progressed. Together, these findings reveal the highly dynamic changes that unfold in a human brain across pregnancy, demonstrating a capacity for extensive neural remodeling well into adulthood.

## Results

### Serological evaluations

**Fig. 1:**
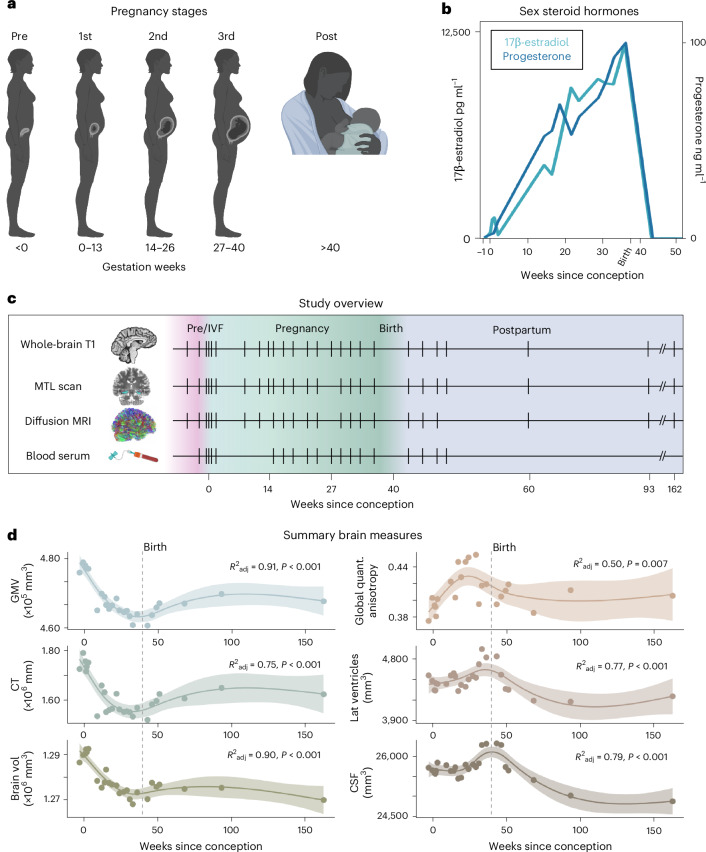
Precision imaging reveals neuroanatomical changes throughout gestation. **a**, Standard medical demarcations for pregnancy stages (that is, trimesters) by gestation week (the image is created with BioRender.com). **b**, Steroid hormones increased significantly throughout pregnancy and dropped precipitously postpartum, as is characteristic of the prenatal and postnatal periods. **c**, A healthy 38-year-old primiparous woman underwent 26 scanning sessions from 3 weeks preconception through 2 years postpartum. Scans were distributed throughout preconception (four scans), first trimester (four scans), second trimester (six scans), third trimester (five scans) and postpartum (seven scans); tick marks indicate when major measures were collected and colors denote pregnancy stage. The participant underwent IVF to achieve pregnancy, allowing for precise mapping of ovulation, conception and gestation week. **d**, Summary (that is, total) of brain measures throughout the experiment. Generalized additive models revealed GMV, CT and total brain volume decreased throughout pregnancy (see Methods for validation with cubic regression), with a slight recovery postpartum. Global QA, lateral ventricle and CSF volumes displayed nonlinear increases across gestation, with a notable rise in the second and third trimesters before dropping sharply postpartum. Shaded regions represent 95% confidence bands; solid lines indicate model fit; dashed line indicates parturition. Source data

Serological evaluations captured canonical hormone fluctuations characteristic of the prenatal, perinatal and postnatal periods (Fig. 1b). Serum hormone concentrations increased significantly over the course of pregnancy and dropped precipitously postpartum (preconception, estradiol (E) = 3.42 pg ml^−1^ and progesterone (P) = 0.84 ng ml^−1^; 3 weeks preparturition, E = 12,400 pg ml^−1^ and P = 103 ng ml^−1^; 3 months postparturition, E = 11.50 pg ml^−1^ and P = 0.04 ng ml^−1^).

### Whole-brain dynamics from baseline through postpartum

To begin, we characterized broad neuroanatomical changes over the course of the entire experimental window (baseline—2 years postpartum, 26 scans; Fig. 1d). Generalized additive models revealed strong nonlinear (effective degrees of freedom > 3) relationships between weeks since conception and summary brain metrics. Total GMV (*F* = 27.87, *P* < 0.001, deviance explained = 93.9%, *R*^2^_adj_ = 0.91), summary CT (*F* = 15.79, *P* < 0.001, deviance explained = 78.6%, *R*^2^_adj_ = 0.75) and total brain volume (*F* = 26.12, *P* < 0.001, deviance explained = 93.4%, *R*^2^_adj_ = 0.90) linearly decreased during gestation and appeared to partially rebound postpartum. In contrast, global microstructural integrity (QA) of white matter increased throughout the first and second trimesters before returning to baseline levels in the postpartum period (whole-brain QA, *F* = 4.62, *P* = 0.007, deviance explained = 60.2%, *R*^2^_adj_ = 0.51). We also observed nonlinear patterns of lateral ventricle expansion *(F* = 10.44, *P* < 0.001, deviance explained = 83.8%, *R*^2^_adj_ = 0.77) and increased cerebrospinal fluid (CSF; *F* = 13.32, *P* < 0.001, deviance explained = 83.8%, *R*^2^_adj_ = 0.79) rising in the second and third trimesters before dropping sharply postpartum.

### Cortical volume and thickness changes tied to gestation

We then narrowed the aperture to capture changes unfolding within gestation itself (baseline—36 weeks pregnant, 19 scans). Relationships between summary brain metrics were evident over the gestational period as follows: total brain volume, GMV and CT were positively associated with one another, whereas lateral ventricles, CSF and global QA demonstrated negative relationships with GMV (Supplementary Fig. 1).

**Fig. 2:**
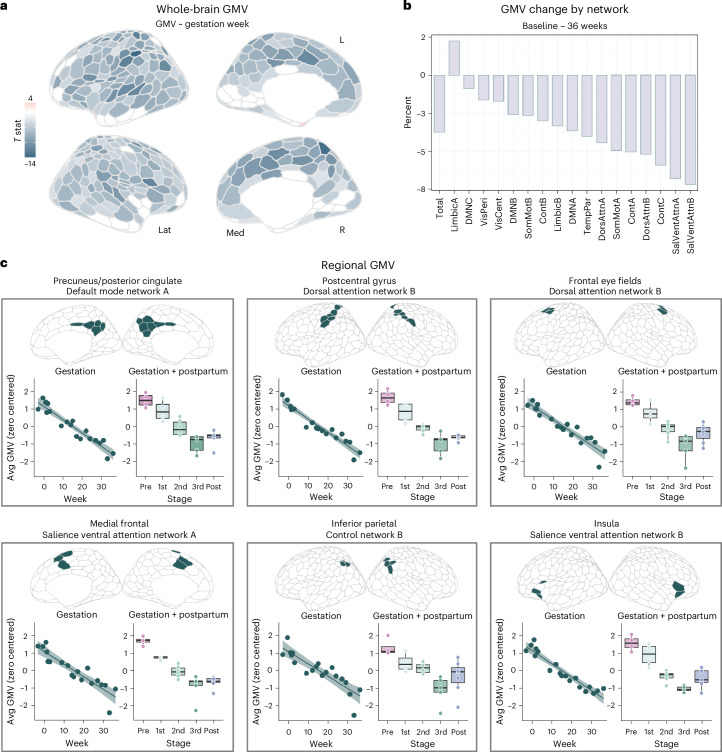
Cortical GMV showed widespread change through gestation and postpartum. **a**, Multivariate regression analyses reveal largely negative relationships between gestation week and regional GMV, with only a minority of regions unaffected or increasing over the gestational window (baseline—36 weeks). All associations presented here were corrected for multiple comparisons (FDR at *q* < 0.05; nonsignificant values set to zero for interpretability). **b**, Average network change was calculated by estimating GMV percent change from baseline (initial) to 36 weeks gestation (final). Attention and control networks appear most affected. **c**, Six representative regions, classified by major subnetworks, that exhibit pronounced GMV change across gestation. For each panel, we display a scatterplot between average GMV of the ROIs and gestation week (left; gestation sessions only, 19 scans), and summary GMV of ROIs by pregnancy stage across the whole study (right; gestation and postpartum sessions, 26 scans). Shaded regions in scatterplots represent a 95% confidence interval. Each boxplot represents IQR for each stage, with a horizontal line representing the median value. The whiskers indicate variability outside (±1.5) of this range. Outside values are >1.5× and <3× IQR beyond either end of the box. All statistical tests were corrected for multiple comparisons (FDR at *q* < 0.05) and values were *z* scored and transformed to have a mean of zero and s.d. of one for easier comparison across regions. Please note that the data values shown here are raw (see Supplementary Tables 1 and 2 and Supplementary Data 1 for exhaustive list). Brain visualizations created with R package ggseg^48^. IQR, interquartile range; Lat, lateral; Med, medial; DMN, default mode network; VisPeri, visual peripheral network; SomMot, somatomotor network; VisCent, visual central network; Cont, control network; TempPar, temporal parietal network; DorsAttn, dorsal attention network; SalVentAttn, salience/ventral attention network. Source data

Changes in GMV were near-ubiquitous across the cortical mantle (Fig. 2a). Most large-scale brain networks exhibited decreases in GMV (Fig. 2b and Supplementary Table 1); indeed, 80% of the 400 regions of interest (ROI) demonstrated negative relationships between GMV and gestation week (Fig. 2a and Supplementary Table 2). Together, these results provide evidence of a global decrease in cortical volume across pregnancy. Several sensory and attention subnetworks were particularly sensitive to gestation, including the control (subnetwork B), salience/ventral attention (subnetwork A), dorsal attention (subnetwork B), default (subnetwork A) and somatomotor (subnetworks A and B) networks (Supplementary Table 1). Regions driving these network-level changes include the bilateral inferior parietal lobe, postcentral gyri, insulae, prefrontal cortex, posterior cingulate and somatosensory cortex (Fig. 2c, Supplementary Table 2 and validation of findings using alternate pipeline in Supplementary Tables 1 and 3). These regions and associated brain networks appear to decrease in volume at a faster rate than the rest of the brain throughout pregnancy, as determined by a subsequent analysis controlling for total GMV (Supplementary Tables 1 and 2). GMV reductions were also significantly correlated with the participant’s estradiol and progesterone concentrations (Supplementary Table 1). A highly similar pattern of results was observed when examining pregnancy-related CT changes (Supplementary Fig. 3 and Supplementary Tables 4 and 5). Significant reductions in cortical GMV over gestation remained after controlling for standard quality control (QC) metrics, albeit with some influence on the magnitude and location of the observed effects (Supplementary Figs. 4 and 5).

In contrast, GMV within regions of the default mode (subnetwork C), limbic (subnetworks A and B) and visual peripheral networks buck the global trend by slightly increasing (for example, temporal poles), remaining constant (for example, orbitofrontal cortex) or reducing at a much slower rate (for example, extrastriate cortex) than total GMV (Fig. 2a,b and Supplementary Tables 1 and 2). CT changes in these regions exhibit similar patterns (Supplementary Fig. 3 and Supplementary Tables 4 and 5).

### Subcortical GMV changes tied to gestation

**Fig. 3:**
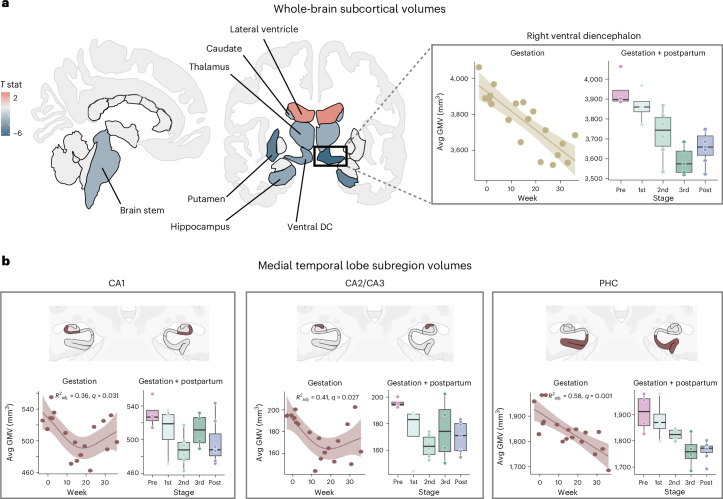
Subcortical GMV changed throughout gestation. **a**, Multivariate regression analyses revealed largely negative relationships between gestation week and subcortical GMV regions over pregnancy, including bilateral thalamus, caudate, hippocampus, ventral diencephalon (encompassing hypothalamus, substantia nigra, mammillary body and red nucleus) and left caudate. Lateral ventricles displayed the only positive relationships with gestation week (also depicted in Fig. 1d). The whole-brain subcortical GMV estimates shown here were derived via FreeSurfer and ‘aseg’ subcortical segmentation. FDR-corrected at *q* < 0.05. Inset, right ventral diencephalon displayed the strongest negative association with gestation (left; baseline—36 weeks, 19 scans) and did not return to baseline postpartum (right; gestation and postpartum, 26 scans). **b**, The participant’s hippocampus and surrounding cortex were segmented into seven bilateral subregions. Quadratic (CA1, CA2/CA3) and linear regression analyses (PHC) revealed subfields were negatively associated with gestation week (baseline—36 weeks, 18 scans) and did not return to baseline postpartum (gestation and postpartum, 25 scans). Shaded regions in scatterplots represent a 95% confidence interval. Each boxplot represents IQR for each stage, with a horizontal line representing the median value. The whiskers indicate variability outside (±1.5) of this range. Outside values are >1.5× and <3× IQR beyond either end of the box. FDR-corrected at *q* < 0.05. For **a** and **b**, nonsignificant regions were set to zero for interpretability. See Supplementary Fig. 6 for complete labeling of regions in both segmentations. Brain visualizations created with R package ggseg^48^*.* DC, diencephalon. Source data

Consistent with the broader cortical reductions in GMV, several subcortical regions significantly reduced in volume across gestation (Fig. 3a, left). This included bilateral ventral diencephalon (right hemisphere values shown in Fig. 3a, right; encompasses hypothalamus, substantia nigra, mammillary body, lateral geniculate nucleus and red nucleus among others^22^), caudate, hippocampus and thalamus, along with left putamen and brain stem (Supplementary Table 6, *q* < 0.05).

Next, high-resolution segmentation of the MTL allowed us to interrogate subcortical structures at a finer resolution, revealing nonlinear volumetric decreases in CA1 (*F*(2,15) = 5.84, *q* = 0.031, *R*^2^_adj_ = 0.36; Fig. 3b, left) and CA2/CA3 (*F*(2,15) = 6.82, *q* = 0.027, *R*^2^_adj_ = 0.41; Fig. 3b, middle) across gestation. PHC exhibited linear volumetric decreases across gestation (*F*(1,16) = 24.87, *q* < 0.001, *R*^2^_adj_ = 0.58; Fig. 3b, right) which was also tied to estradiol (*F*(1,12) = 20.21, *q* = 0.005, *R*^2^_adj_ = 0.60). All three relationships remained significant after proportional correction for total GMV. There was no significant change in other subregions or total volume of the hippocampal body, or in the parahippocampal gyrus (Supplementary Table 7 and Supplementary Fig. 8).

### White matter microstructure changes tied to gestation

**Fig. 4:**
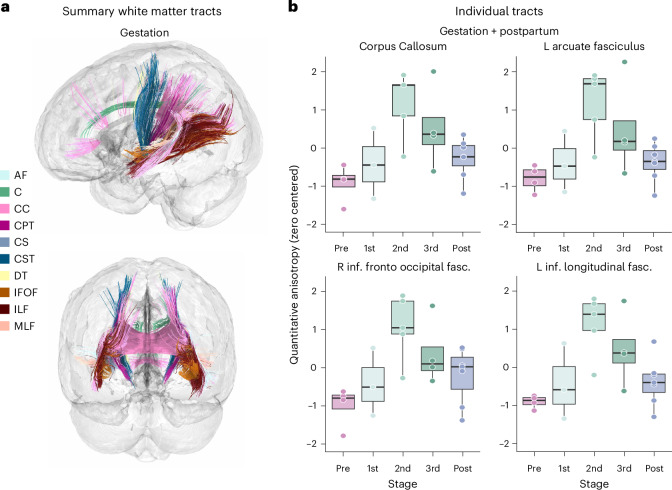
White matter microstructure changes throughout the experiment. **a**, Numerous white matter tracts demonstrate increasing QA in relation to advancing gestation week (baseline—36 weeks, 16 scans), as determined by correlational tractography analysis (FDR, *q* < 0.0001). See Supplementary Table 9 for complete list of tracts with a significant correlation between QA and gestation week. **b**, Summary of QA values by pregnancy stage (gestation and postpartum, 23 scans) for representative ROIs significantly tied to gestation. ROI-based tractometry was used to extract QA values. Each boxplot represents IQR for each stage, with a horizontal line representing the median value. The whiskers indicate variability outside (±1.5) of this range. Outside values are >1.5× and <3× IQR beyond either end of the box. Values were *z* scored and transformed to have a mean of zero and s.d. of one for easier comparison across individual tracts. AF, arcuate fasciculus; C, cingulum bundle; CC, corpus callosum; CPT, corticopontine tracts; CS, corticostriatal tracts; CST, corticospinal tracts; DT, dentatorubrothalamic tract; IFOF, inferior frontal occipital fasciculus; ILF, inferior longitudinal fasciculus; MLF, middle longitudinal fasciculus. Source data

In contrast to decreasing global GMV, correlational tractography of white matter, which tests for linear trends in the data, revealed increasing microstructural integrity across the whole brain during gestation (Fig. 4a), concomitant with the rise in 17β-estradiol and progesterone (all *q* < 0.001; Supplementary Fig. 9). Tracts displaying robust correlations with gestational week included the corpus callosum, arcuate fasciculus, inferior fronto-occipital fasciculus and inferior longitudinal fasciculus (Fig. 4b), as well as the cingulum bundle, middle and superior longitudinal fasciculus, corticostriatal, corticospinal and corticopontine tracts (see Supplementary Table 9 for complete list).

### Comparing brain changes across pregnancy against controls

We then compared the changes in GMV across gestation to that of typical variability over time, derived from eight densely-sampled controls^23^. The GMV changes we see across pregnancy far exceed normative brain variability (Supplementary Fig. 11). On average, change in cortical GMV was nearly three times higher than controls scanned over a similar duration (Supplementary Fig. 11a,b). This extends to MTL subfields, wherein change in volume was three to four times greater across gestation than normative brain variability (Supplementary Fig. 11c,d). We contextualized these findings further by comparing gestational GMV change against our participant’s preconception brain volumes; average GMV change during pregnancy was six times (cortical) and three times (MTL) higher than the variability observed between baseline sessions.

## Discussion

Converging evidence across mammalian species points to pregnancy as a remarkable period of neuroplasticity, revealing the brain’s ability to undergo adaptive, hormonally-driven neuroanatomical changes beyond adolescence^13–15,20,21,24–26^. Investigations that compare women prepregnancy and then again postpartum provide the strongest evidence to date that the human brain undergoes such neural changes^11,27^. But what about pregnancy itself? Over what time course do anatomical changes in the maternal brain manifest? Are they tied to the substantial increase in sex hormone production? Here we begin to address these outstanding questions. This study and corresponding open-access dataset offer neuroscientists a detailed map of the human brain across gestation, a resource for which a wide range of previously unattainable neurobiological questions can now be explored.

Our findings from this precision imaging study show that pregnancy is characterized by reductions in GMV, cortical thinning and enhanced white matter microstructural integrity that unfold week by week. These changes were also tied to the significant rise in steroid hormone concentrations over pregnancy. Some of these changes persist at 2 years postpartum (for example, global reductions in GMV and CT), while others, including markers of white matter integrity, appear to be transient. Ventricular expansion and contraction parallel these cortical changes. These widespread patterns, and the notable increase in CSF volume across gestation, could reflect increased water retention and subsequent compression of cortical tissue. However, the persistence of these changes at 2 years postpartum and regional variation in GMV, CT and QA, hint at cellular underpinnings, such as alterations in glia or neuron number, synaptic density and myelination (for review on the latter, see ref. ^4^). Future studies of the relationship between fluid dynamics and volumetric changes will help clarify the factors that drive global neural changes during pregnancy; such insights will have broad implications for maternal health (for example, neurological effects tied to pre-eclampsia or edema).

Critically, dynamic neural changes occurred within the pregnancy window itself, a nuance not captured by studies limited to comparisons between prepregnancy and postpregnancy. For example, we observed large increases in white matter microstructural integrity (QA) throughout the first and second trimesters of pregnancy, but these measures fully returned to baseline values by the first postpartum scan. This pattern may explain why previous studies report no pregnancy-related differences in white matter tractography^14^. Other measures, such as GMV and CT, decreased throughout gestation and displayed only a modest rebound postpartum. These nonlinear patterns suggest that only quantifying prepregnancy and postpartum brain structure may overlook the full range of changes that unfold within the gestational window, and underrepresent the brain’s metamorphosis during pregnancy. Furthermore, although observed changes were largely global, some regions displayed notable stability (for example, extrastriate cortex). The subcortical region that displayed the strongest relationship with gestation week was the ventral diencephalon, which encompasses the hypothalamus and subsequent medial preoptic area and paraventricular nucleus—structures critical for inducing maternal behavior^12,16^. The hippocampus exhibited a reduction in volume across gestation, and with higher spatial resolution, this reduction was revealed to be driven by changes in CA1 and CA2/CA3 subfield volumes, while other hippocampal subfields remained stable. Adjacent PHC within the MTL also exhibited volume reduction across gestation. While our hippocampal findings are consistent with pre/post studies of pregnancy^13^, the precision lens applied within gestation revealed the nonlinear nature of this reduction. Recapitulating and clarifying these regionally specific patterns of volume change throughout the MTL merits further investigation.

Similar precision imaging studies have captured dynamic brain reorganization across other neuroendocrine transitions, such as the menstrual cycle (see review in ref. ^28^), underscoring the powerful role steroid hormones have in shaping the mammalian brain^29^. Endocrine changes across pregnancy dwarf those that occur across the menstrual cycle, which highlights the critical need to map the brain’s response to this unique hormonal state. Broad physiological changes occur in tandem with the rise in steroid hormones, including changes in body mass composition, water retention, immune function and sleep patterns^11^. These factors could have a role in the brain changes observed here, with some driving neurobiological changes and others, like water retention, potentially affecting MRI-based measurements. Note that, although cortical reductions in GMV over gestation were stable across analyses, accounting for QC measures influenced the magnitude and location of these results. These metrics all fell within the standard range, but there may be meaningful reductions in signal that accompany volumetric reductions (for example, increased CSF and decreased GM)—a methodological nuance that goes beyond the scope of this resource study. Ultimately, identifying the shared and unique contributions of these factors to the neuroanatomical changes that unfold across gestation warrants further investigation. Deeply phenotyping a large and diverse cohort of women across pregnancy will open up new avenues of exploration, for example, allowing researchers to link blood-based proteomic signatures to pregnancy outcomes; deploying wearable devices to monitor changes in sleep, cognition and mood; and probing the broader social and environmental determinants of maternal health^27^.

The neuroanatomical changes that unfold during matrescence may have broad implications for understanding individual differences in parental behavior^13,24,30,31^, vulnerability to mental health disorders^32,33^ and patterns of brain aging^18,19,34–36^. Decreases in GMV may reflect ‘fine-tuning’ of the brain by neuromodulatory hormones in preparation for parenthood^26^. For example, in rodents, steroid hormones promote parental behavior by remodeling specific neural circuits in the medial preoptic area of the hypothalamus. These behavioral adaptations are critical to the dam’s ability to meet the demands of caring for the offspring^12^. Human studies have revealed GMV reductions in areas of the brain important for social cognition and the magnitude of these changes corresponds with increased parental attachment^13^. Deeper examination of cellular and systems-level mechanisms will improve our understanding of how pregnancy remodels specific circuits to promote maternal behavior.

Although studied to a lesser degree, ties between maternal behavior and white matter microstructure (particularly connectivity between temporal and occipital lobes) have been noted^31^. Here we reveal pronounced GMV changes in regions within sensory, attention and default mode networks over the gestational window. In parallel, we observed increased anisotropy in white matter tracts that facilitate communication between emotional and visual processing hubs^37–39^, including the inferior longitudinal fasciculus and inferior fronto-occipital fasciculus. Pinpointing the synchrony of gray and white matter changes that unfold in the maternal brain could be key to understanding the behavioral adaptions that emerge during and after pregnancy, such as honing the brain’s visual and auditory responses to infant cues and eliciting maternal behavior. Research into other major transition periods supports this idea. For instance, adolescence is a dynamic period characterized by region-specific, nonlinear decreases in GMV and increases in WMV, maturational brain changes that are tied to gains in executive function and social cognition^40^. For both adolescence^41^ and matrescence, the considerable rise in steroid hormone production appears to remodel the brain (see ref. ^25^ for comparative analysis), promoting a suite of behaviors adaptive to that life stage. How specific neural changes give rise to specific behavioral adaptations has yet to be fully explored with respect to human pregnancy.

This precision imaging study mapped neuroanatomical changes across pregnancy in a single individual, precluding our ability to generalize to the broader population. To benchmark our findings, we compared the magnitude of GMV changes observed throughout pregnancy against data from nonpregnant individuals sampled over a similar time course. Doing so provided compelling evidence that pregnancy-related neuroanatomical shifts far exceed normative day-to-day brain variability and measurement error. Evidence suggests that white matter microstructure remains fairly stable over a six-month period^42^, but more studies are needed to compare the degree of white matter changes observed during pregnancy to normative change over time. Further, sampling larger cohorts of women will generate much-needed normative models of brain change (akin to ref. ^43^) throughout pregnancy to establish what constitutes a typical degree of neuroanatomical change expected during gestation and postpartum recovery.

These findings provide a critical rationale for conducting further precision imaging studies of pregnancy in demographically enriched cohorts to determine the universality and idiosyncrasy of these adaptations and their role in maternal health. Are the changes observed in our participant reflective of the broader population? Do deviations from the norm lead to maladaptive outcomes? A precision imaging approach can help determine whether the pace of pregnancy-induced neuroanatomical changes drives divergent brain health outcomes in women, as may be the case during other rapid periods of brain development^44^. One in five women experiences perinatal depression^45^ and while the first FDA-approved treatment is now available^46^, early detection remains elusive. Precision imaging studies could offer clues about an individual’s risk for or resilience to depression before symptom onset, helping clinicians better determine when and how to intervene. Neuroscientists and clinicians also lack tools to facilitate detection and treatment of neurological disorders that co-occur, worsen or remit with pregnancy, such as epilepsy, headaches, multiple sclerosis and intracranial hypertension^47^. Precision mapping of the maternal brain lays the groundwork for a greater understanding of the subtle and sweeping structural, functional, behavioral and clinical changes that unfold across pregnancy. Such pursuits will advance our basic understanding of the human brain and its remarkable ability to undergo protracted plasticity in adulthood.

## Methods

### Participant

Our participant (E.R.C.) was a healthy 38-year-old primiparous woman who underwent in-vitro fertilization (IVF) to achieve pregnancy. Previous studies reported no observable differences in neural changes from prepregnancy to postpregnancy between women who conceived naturally versus women who conceived via IVF^13^, and doing so provides a controlled way of monitoring pregnancy status. The participant experienced no pregnancy complications (for example, gestational diabetes and hypertension), delivered at full term via vaginal birth, nursed through 16 months postpartum, and had no history of neuropsychiatric diagnosis, endocrine disorders, prior head trauma or history of smoking. The participant gave written informed consent and the study was approved by the University of California, Irvine Human Subjects Committee.

### Study design

The participant underwent 26 MRI scanning sessions from 3 weeks before conception through 2 years postpartum (162 weeks), during which high-resolution anatomical and diffusion spectrum imaging scans of the brain were acquired. Scans were distributed throughout this period, including prepregnancy (four scans), first trimester (four scans), second trimester (six scans), third trimester (five scans) and postpartum (seven scans; Fig. 1c). The first 6 sessions took place at the UCSB Brain Imaging Center (BIC), the final 20 sessions took place at the UCI Facility for Imaging and Brain Research (FIBRE). The majority of scans took place between 9 AM and 2 PM, limiting significant AM–PM fluctuations^49^. The MRI protocol, scanner (Siemens 3T Prisma) and software (version MR E11) were identical across sites. Each scanner was checked weekly for the duration of the study and passed all QC reports indicating no significant alterations in the geometry. To ensure the robustness of the findings, after the final study session, the participant completed back-to-back validation scans at UCI and UCSB within a 12-h window to assess reliability between scanners. Intraclass correlation coefficients (two-way, random effects, absolute agreement, single rater) reveal ‘excellent’ test–retest reliability between scanners, including ROI-level GMV (ICC = 0.97, 95% CI: 0.80–0.99), ROI-level CT (ICC = 0.96, 95% CI: 0.90–0.98), MTL subfield volume (ICC = 0.99, 95% CI: 0.97–0.99) and ROI-level QA (ICC = 0.94, 95% CI: 0.91–0.97). Furthermore, when examining the relationship between gestation week and GMV among UCI-only gestational sessions, findings were consistent (Supplementary Fig. 12), indicating that site differences are highly unlikely to have contributed meaningfully to the observed effects. Although not applicable here, we note that having a control participant scanned over a similar duration within the same scanner is critical for estimating how much variation in the brain can be attributed to within-scanner variability.

To monitor state-dependent mood and lifestyle measures, the following scales were administered on each experiment day: Perceived Stress Scale^50^, Pittsburgh Sleep Quality Index^51^, State-Trait Anxiety Inventory for Adults^52^ and Profile of Mood States^53^. Correlation analyses between state-dependent measures, summary brain metrics and gestation week revealed little to no relationships. The only exception to this was a moderate negative association between global QA and state anxiety (Spearman’s correlation (*ρ*) = −0.65, *q* = 0.04; baseline—36 weeks, *n* = 16). By making this data openly accessible, we encourage a more nuanced approach toward exploring mood and lifestyle measures in relation to brain changes over pregnancy.

### Endocrine procedures

The participant underwent a blood draw (*n* = 19; Fig. 1c) before MRI scanning. Sex steroid concentrations were determined via ultra-sensitive liquid chromatography–mass spectrometry at the Brigham and Women’s Hospital Research Assay Core (BRAC). Assay sensitivities, dynamic range and intra-assay coefficients of variation were as follows: estradiol—1.0 pg ml^−1^, 1–500 pg ml^−1^, <5% relative s.d. (RSD); progesterone—0.05 ng ml^−1^, 0.05–10 ng ml^−1^, 9.33% RSD. Serological samples were not acquired in five sessions due to scheduling conflicts with UC Irvine’s Center for Clinical Research.

#### MRI acquisition

MRI scanning sessions at the University of California, Santa Barbara and Irvine were conducted on 3T Prisma scanners equipped with 64-channel phased-array head/neck coil (of which 50 coils are used for axial brain imaging). High-resolution anatomical scans were acquired using a T1-weighted (T1w) magnetization prepared rapid gradient echo (MPRAGE) sequence (repetition time (TR) = 2,500 ms, time to echo (TE) = 2.31 ms, inversion time (TI) = 934 ms, flip angle = 7°, 0.8 mm thickness) followed by a gradient echo field map (TR = 758 ms, TE1 = 4.92 ms, TE2 = 7.38 ms, flip angle = 60°). A T2-weighted (T2w) turbo spin echo scan was also acquired with an oblique coronal orientation positioned orthogonally to the main axis of the hippocampus (TR/TE = 9,860/50 ms, flip angle = 122°, 0.4 × 0.4 mm^2^ in-plane resolution, 2-mm slice thickness, 38 interleaved slices with no gap, total acquisition time = 5 min and 42 sec). The Diffusion Spectrum Imaging (DSI) protocol sampled the entire brain with the following parameters: single phase, TR = 4,300 ms, echo time = 100.2 ms, 139 directions, *b*-max = 4,990, FoV = 259 × 259 mm, 78 slices, 1.7986 × 1.7986 × 1.8 mm voxel resolution. These images were linearly registered to the whole-brain T1w MPRAGE image. A custom foam headcase was used to provide extra padding around the head and neck, as well as to minimize head motion. Additionally, a custom-built sound-absorbing foam girdle was placed around the participant’s waist to attenuate sound near the fetus during second-trimester and third-trimester scanning.

#### Image processing

##### Cortical volume and thickness

CT and GMV were measured with Advanced Normalization Tools^54^ version 2.1.0 (ANTs). We first built a subject-specific template (SST) (antsMultivariateTemplateConstruction2) and tissue priors (antsCookTemplatePriors) based on our participant’s two preconception whole-brain T1-weighted scans to examine neuroanatomical changes relative to the participant’s prepregnancy baseline. We used labels from the OASIS population template, provided by ANTs, as priors for this step. For each session, the structural image was processed and registered to the SST using the ANTs CT pipeline (antsCorticalThickness). This begins with an N4 bias field correction for field inhomogeneity, then brain extraction using a hybrid registration/segmentation method^55^. Tissue segmentation was performed using Atropos^54^ to create tissue masks of CSF, gray matter, white matter and deep gray matter. Atropos allows prior knowledge to guide the segmentation algorithm, and we used labels from our SST as priors to minimize warping and remain in native participant space. CT measurements were then estimated using the DiReCT algorithm^56^, which estimates the gray–white matter interface and the gray matter–CSF interface and computes a diffeomorphic mapping between the two interactions, from which thickness is derived. Each gray matter tissue mask was normalized to the template and multiplied to a Jacobian image that was computed via affine and nonlinear transforms. Using MATLAB (version 2022a), summary, regional-level estimates of CT, GMV and CSF for each scan were obtained by taking the first eigenvariate (akin to a ‘weighted mean’^57^) across all voxels within each parcel of the Schaefer 400-region atlas^58^. We then averaged ROIs across networks, which were defined by the 17-network Schaefer scheme^58,59^. Global measures of CT, GMV and CSF were computed for each session by summing across all voxels within the respective output image; total brain volume was computed by summing across all voxels within each session’s brain extraction mask. Our findings held when using an SST derived from all 26 MRIs (prepregnancy through postpartum), as well as when estimating the mean (versus weighted mean) of all voxels within each parcel. The ANTs CT pipeline is highly validated with good test–retest reproducibility and improved ability to predict variables such as age and gender from region-wise CT measurements compared to surface-based FreeSurfer^55^. However, to reproduce our findings across software packages, we also ran the T1w data through the longitudinal FreeSurfer (v.7) CT pipeline^60,61^, which corroborated our findings using both the Schaefer-400 (Supplementary Fig. 2 and Supplementary Tables 1 and 4) and popular Desikan–Killiany^62^ (Supplementary Table 3) cortical parcellations. Whole-brain T1w-based subcortical volume estimates (including cerebellum and lateral ventricles) were also derived using this FreeSurfer pipeline, wherein we derived 28 region-of-interest estimates via the commonly used ‘aseg’ parcellation scheme^63^ (Supplementary Fig. 6a). A complete reporting of findings can be found in Supplementary Data 1.

Mean framewise displacement (FWD) estimates from gestation sessions with a 10-min resting-state scan (*n* = 18) were used to indirectly assess whether motion increased throughout pregnancy. Average FWD (mm) was extremely minimal across the entire experiment (*M* = 0.13, s.d. = 0.02, range = 0.09–0.17) and varied only slightly by pregnancy stage (pre, *M* = 0.11 and s.d. = 0.004; first, *M* = 0.11 and s.d. = 0.01; second, *M* = 0.13 and s.d. = 0.02; third, *M* = 0.16 and s.d. = 0.007; post, *M* = 0.13 and s.d. = 0.01). While mean FWD did correspond with gestation week (*r* = 0.88, *P* < 0.001), controlling for this did not alter our main findings (for example, total GMV remained negatively associated with gestation after partial correlation with FWD (*r* = −0.87 and *P* < 0.001) because motion differences between stages were minuscule (Supplementary Fig. 4a).

As a further test of the robustness of the dataset, we ran QC assessments on all T1w images using the IQMs pipeline^64^ from MRIQC (version 23.1). Assessments of interest included (1) coefficient of joint variation (CJV), (2) signal-to-noise ratio for gray matter (SNR) and (3) contrast-to-noise ratios (CNR). All QC metrics fell within expected standard ranges^65^ (Supplementary Fig. 4b–d). Although relationships existed between gestation week and QC measures (CJV, *r* = 0.70 and *P* < 0.001; SNR and CNR, *r* = −0.83 and *P* < 0.001), including these variables in the regression models did not detract from our finding suggesting cortical GMV reductions occur over gestation, especially within regions belonging to attention and somatosensory networks (Supplementary Fig. 5). When looking across all MRIQC outputs, discrepancies were noted in session seven (gestation week nine, first trimester). Removing this day from the analyses only strengthened observed relationships between cortical volume and gestation; however, for completeness, data from this day is included in the main findings. These QC outputs for each session of the experiment can be found in Supplementary Data 1. Finally, we used FreeSurfer’s Eueler number to evaluate a field-standard quantitative assessment of each T1w structural image^66^. We observed no significant relationships between the Euler number and gestation week or summary brain metrics. A discrepancy (for example, two s.d. below average) was noted in session eight; however, again, removing this session did not detract from our main findings showing reductions in GMV over gestation.

##### Hippocampal segmentation

T1- and T2-weighted images (*n* = 25) were submitted to the automatic segmentation of hippocampal subfields package (ASHS^67^, version July 2018) for parcellation of seven MTL subregions: CA1, CA2/CA3, dentate gyrus, subiculum, perirhinal cortex, entorhinal cortex and PHC (Supplementary Fig. 6b). The ASHS segmentation pipeline automatically segmented the hippocampus in the T2w MRI scans using a segmented population atlas, the Princeton Young Adult 3T ASHS Atlas template^68^ (*n* = 24, mean age = 22.5 years). A rigid-body transformation aligned each T2w image to the respective T1w scan for each day. Using ANTs deformable registration, the T1w was registered to the population atlas. The resulting deformation fields were used to resample the data into the space of the left and right template MTL ROI. Within each template ROI, each of the T2w scans of the atlas package was registered to that day’s T2w scan. The manual atlas segmentations were then mapped into the space of the T2w scan, with segmentation of the T2w scan computed using joint label fusion^69^. Finally, the corrective learning classifiers contained in ASHS were applied to the consensus segmentation produced by joint label fusion. The output of this step is a corrected segmentation of the T2w scan. Further description of the ASHS protocol can be found here^67^. T2w scans and segmentations were first visually examined using ITK-SNAP^70^ for quality assurance and then subjected to manual editing in native space using ITK-SNAP (v.3.8.0-b; C.M.T.). One session (scan 15, third trimester) was discarded due to erroneous scan orientation. The anterior extent of the segmented labels was anchored 4 mm (two slices) anterior to the appearance of the limen insulae, and the posterior extent was anchored to the disappearance of hippocampal gray matter from the trigone of the lateral ventricle. Boundaries between perirhinal, entorhinal and parahippocampal cortices were established in keeping with the Olsen–Amaral–Palombo (OAP) segmentation protocol^71^. In instances where automatic segmentation did not clearly correspond to the underlying neuroanatomy, such as when a certain label was missing several gray matter voxels, manual retouching allowed for individual voxels to be added or removed. All results are reported using the manually retouched subregion volumes to ensure the most faithful representation of the underlying neuroanatomy. Scans were randomized and segmentation was performed in a random order, blind to pregnancy stage. To assess intrarater reliability for the present analyses, two days underwent manual editing a second time. The generalized Dice similarity coefficient^72^ across subregions was 0.87 and the intraclass correlation coefficient was 0.97, suggesting robust reliability in segmentation.

##### White matter microstructure

Diffusion scans were preprocessed using the automation software QSIprep (version 0.16.1) compiled using a singularity container^73^ and run primarily with the default parameters, with the exceptions ‘–output resolution 1.8’, ‘–dwi denoise window 5′,–force-spatial-normalization’, ‘–hmc model 3dSHORE’, ‘–hmc-transform Rigid’ and ‘–shoreline iters 2’. Twenty-three sessions were preprocessed and analyzed, with the remaining three scans excluded due to missing DSI scans (sessions 9 and 15) or corresponding field map for distortion correction (session 7). Despite passing QC assessments during preprocessing, visual inspection of the field maps in session 10 revealed a slight artifact. However, removal of this session had minimal impact on the overall results and remained in the final analyses. T1w images were corrected for intensity nonuniformity (N4BiasFieldCorrection) and skull-stripped (antsBrainExtraction). The images underwent spatial normalization and registration to the ICBM 152 Nonlinear Asymmetric template. Finally, brain tissue segmentation of CSF, GM and WM was performed on each brain-extracted T1w using FMRIB’s Automated Segmentation Tool (FAST). Preprocessing of diffusion images began by implementing MP-PCA denoising with a 5-voxel window using MRtrix3’s dwidenoise function. B1 field inhomogeneity was corrected using dwibiascorrect from MRtrix3 with the N4 algorithm. Motion was corrected using the SHORELine method. Susceptibility distortion correction was based on GRE field maps. Preprocessed Nifti scans were prepared for tractography using DSI Studio via singularity container version Chen-2022-07-31 (ref. ^74^). Diffusion images were converted to source code files using the DSI Studio command line ‘--action=src’ and a custom script to convert all images. The diffusion data were reconstructed in MNI space using q-space diffeomorphic reconstruction^75^ with a diffusion sampling of 1.25 and output resolution of 1.8 mm isotropic. The following output metrics were specified to be included in the output FIB file: QA and mean diffusivity (MD). The quality and integrity of reconstructed images were assessed using ‘QC1: SRC Files Quality Control’. First, the consistency of image dimension, resolution, DWI count and shell count was checked for each image. Second, each image was assessed for the ‘neighboring DWI correlation’ which calculates the correlation coefficient of low *b* DWI volumes that have similar gradient direction. Lower correlation values may indicate issues with the diffusion signal due to artifacts or head motion. Finally, DSI Studio performed an outlier check, labeling images as a ‘low-quality outlier’ if the correlation coefficient was >3 s.d. from the absolute mean. None of our scans were flagged as outliers. The reconstructed participant files were aggregated into one connectometry database per metric.

##### Day2Day control dataset

To compare our findings against a control group of nonpregnant densely-sampled individuals, we used the Day2Day dataset^23^ which offered comparable whole-brain T1 and T2 MTL scans for eight participants (two male) scanned 12–50 times over 2–7 months. Each participant was run through the ANTs CT and ASHS processing pipelines as outlined above (‘Cortical volume and thickness’ and ‘Hippocampal segmentation’). To note, for each participant, we created an SST based on their first two sessions for consistency with the primary dataset; subfield volumes for the T2 MTL scans did not undergo manual retouching. Due to missing header information on the publicly available diffusion scans, we were unable to benchmark our white matter changes with the Day2Day dataset.

#### Statistical analysis

Statistical analyses were conducted using R (sMRI; version 3.4.4) and DSI Studio (dMRI; Chen-2022-07-31).

##### Summary brain metrics

To reflect the existing literature, we first explored brain metrics across the entire study duration (prepregnancy through postpartum, *n* = 26 scans). When including all sessions, total brain volume, GMV, CT, global QA, ventricle volume and CSF displayed nonlinear trends over time; therefore, we used generalized additive models (GAM; cubic spline basis, *k* = 10, smoothing = GCV), a method of nonparametric regression analysis (R package, mgcv^76^), to explore the relationship between summary brain metrics (outcome variables) and gestation week (smooth term). Each model underwent examination (gam.check function) to ensure it was correctly specified with regards to (1) the choice of basis dimension (*k*) and (2) the distribution of model residuals (see mgcv documentation in ref. ^76^). The general pattern of results held after toggling model parameters; however, we note the risk of overinterpreting complex models with small sample sizes^77^. To address overfitting and cross-validate our basis type selection, we also fit the data using nonpenalized general linear models (GLM) with both linear and polynomial terms for gestation week. We compared the performance of each GLM (that is, models using only a linear term versus models with polynomial terms) via the Akaike information criterion (AIC), which revealed that cubic models consistently outperformed both linear and quadratic models (AIC_diff_ > 3), providing additional evidence for nonlinear changes in structural brain variables over time. Determining whether these patterns replicate in larger cohorts and whether complex models are better suited to capture data patterns across individuals will be a necessary next step.

##### Cortical GMV and CT

We then narrowed our analyses to the first 19 sessions (baseline—36 weeks gestation) to assess novel brain changes occurring over the gestational window. We first computed Pearson’s product-moment correlation matrices between the following variables: gestation week, estradiol, progesterone and the 17 network-level average GMV values. We then ran a multivariate regression analysis predicting ROI-level GMV changes by gestation week. To identify which regions were changing at a rate different from the global decrease, we then ran the analyses again to include total GMV in the regression model (Supplementary Table 2). This was extended to the network level, where we ran partial correlations accounting for total GMV. These same analyses were then run with CT measures. Globally-corrected results provided in Supplementary Tables 1–5. Percent change at the network level was computed by subtracting the final pregnancy value (36 weeks pregnant) from the first prepregnancy baseline value, then dividing that difference by said first prepregnancy baseline value. All analyses underwent multiple comparisons testing (false discovery rate (FDR)-corrected at *q* < 0.05).

##### Subcortical GMV

A similar statistical approach was taken for subcortical volume estimates. We ran a multivariate regression analysis predicting GMV changes over gestation in 28 ROIs (Supplementary Fig. 6a) by gestation week (FDR-corrected at *q* < 0.05).

To evaluate the relationship between gestation week and MTL subregion volume over pregnancy (*n* = 7 bilateral subregions and *n* = 18 MTL scans), we used a combination of linear and nonlinear models based on individual subregion data patterns. Models were compared for best fit with each subregion via AIC from the GLM output (as described in ‘Summary brain metrics’). A linear regression model was most appropriate for PHC (AIC_diff_ < 3), whereas a quadratic model performed best for CA1 and CA2/CA3. As a control, we repeated the analyses with MTL subregion volumes after proportional volume correction of total GMV calculated by ASHS. Finally, we evaluated the relationship between endogenous sex hormones (estrogen and progesterone) and subregion volumes using linear regression. Relationships were considered significant only if they met FDR correction at *q* < 0.05.

##### White matter microstructure

DSI Studio’s correlational tractography^74^ was used to analyze the relationship between white matter structure and gestational week (*n* = 16). A truncated model was run to examine the relationship between white matter and sex steroid hormones (*n* = 14) for the subset of diffusion scans with paired endocrine data during gestation. A nonparametric Spearman’s correlation was used to derive the correlation between gestational week and endocrine factors and our metrics of interest (QA and MD; see Supplementary Table 9 and Supplementary Fig. 10 for MD results) because the data were not normally distributed. Statistical inference was reached using connectometry, a permutation-based approach that tests the strength of coherent associations found between the local connectome and our variables of interest. It provides higher reliability and replicability by correcting for multiple comparisons. This technique provides a high-resolution characterization of local axonal orientation. The correlational tractography was run with the following parameters: *t* score threshold of 2.5, four pruning iterations and a length threshold of 25 voxel distance. To estimate the FDR, a total of 4,000 randomized permutations were applied to obtain the null distribution of the track length. Reported regions were selected based on FDR cutoff (FDR < 0.2, suggested by DSI Studio), and contained at least ten tracts. For visualization of global and tract QA at each gestational stage, mean QA values were extracted using DSI Studio’s whole-brain fiber tracking algorithm and ROI-based tracking using the default HCP842 atlas^78^.

##### Day2Day dataset: measurement variability

To establish a marker of normative variability over half a year, we computed metrics of measurement variability using the Day2Day dataset^23^, which provided both whole-brain T1 and high-resolution T2 MTL scans. For each region, *j*, of the Schaefer parcellation, we assessed across-session variability, *ε*, as

$${\varepsilon }_{j}=100\times {\rm{mean}}\left(\frac{\left|{t}_{s}-\hat{t}\right|}{\hat{t}}\right)$$

Where *t*_*s*_ is the morphometric measurement of a parcel for session *s* and $\hat{t}$ is the mean of *t* across sessions^55,79^. Thus, we defined variability as the mean absolute percent difference between each individual and the mean across sessions. Across-session variability estimates for all 400 regions were then averaged across eight participants, and a global measure of cortical GMV variability was computed by averaging across the 400 regions. This approach was repeated independently for the T2 hippocampal scans, wherein we computed across-session variability for each parcel of the ASHS parcellation scheme (*n* = 7 bilateral subfields). However, it is important to note that raw subfield values (that is, no manual retouching) were used for Day2Day variability assessments and should be interpreted with caution. Finally, to better compare against our own data, we repeated this approach using our participant’s first two baseline scans (that is, preconception) to derive within-participant variability estimates.

Benchmarking our data in this way allows us to capture the degree of change expected due to factors such as image processing and instrumentation variability or other day-to-day changes that could potentially modulate brain size and shape (see ref. ^80^ for review). The percent change observed over pregnancy (baseline versus 36 weeks gestation) far exceeds the expected variability estimated using both the Day2Day dataset (Supplementary Fig. 11) and our within-participant control data. This was quantified by dividing the observed percent change in GMV metrics (baseline versus 36 weeks) by the global measure of GMV percent variability of each control group (that is, Day2Day, within-participant control), independently for cortex and subcortex.

### Reporting summary

Further information on research design is available in the Nature Portfolio Reporting Summary linked to this article.

## Online content

Any methods, additional references, Nature Portfolio reporting summaries, source data, extended data, supplementary information, acknowledgements, peer review information; details of author contributions and competing interests; and statements of data and code availability are available at 10.1038/s41593-024-01741-0.

## Supplementary information

Supplementary Information Supplementary Figs. 1–12 and Supplementary Tables 1–10.

Reporting Summary

Supplementary Data 1 Supporting data for primary analyses and quality control reports.

Supplementary Data 2 Supporting data for Supplementary Figs. 1, 3–5, 7, 8, 11 and 12.

## Source data

Source Data Fig. 1 Statistical source data.

Source Data Fig. 2 Statistical source data.

Source Data Fig. 3 Statistical source data.

Source Data Fig. 4 Statistical source data.

## Data Availability

The dataset consists of 26 MRI scans (T1w, T2w and diffusion scans) alongside state-dependent measures and serum assessments of ovarian sex hormones for each session. The raw data is publicly available at https://openneuro.org/datasets/ds005299. Source data are provided with this paper.

## Code availability

No custom code was used.
