# Supplementary material for: Neuroanatomical changes observed over the course of a human pregnancy
Source: Nat Neurosci. 2024 Sep 16;27(11):2253–60. doi: 10.1038/s41593-024-01741-0 (PMC11537970; doi:10.1038/s41593-024-01741-0)
Supplement: Supplementary file 1 — Supplementary Figs. 1–12 and Supplementary Tables 1–10. [file 41593_2024_1741_MOESM1_ESM.pdf]

nature neuroscience

Supplementary information https://doi.org/10.1038/s41593-024-01741-0

Neuroanatomical changes observed over the course of a human pregnancy

In the format provided by the authors and unedited

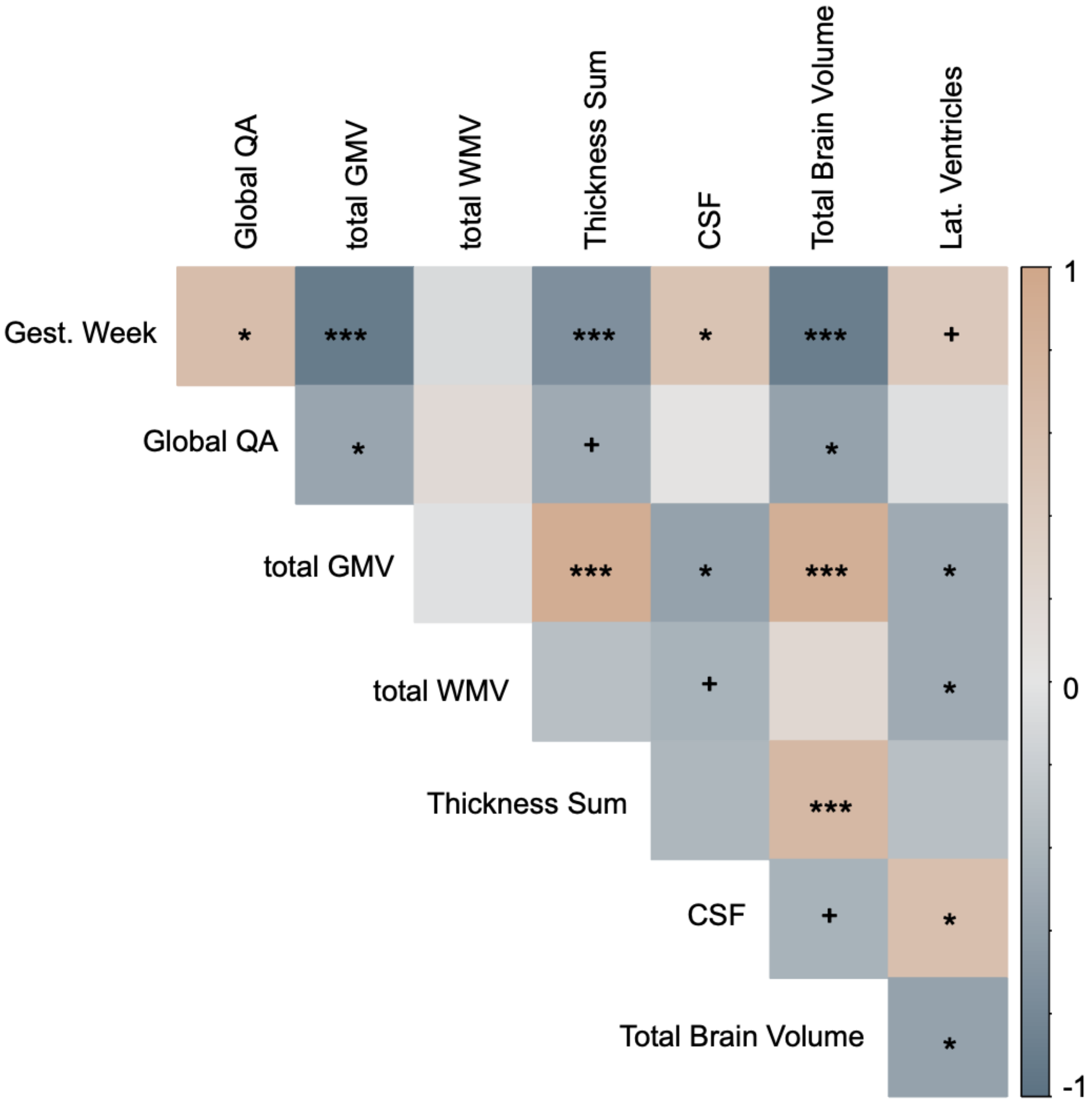

Supplementary Figure 1. Relationships between summary (i.e., total) brain measures and gestation week across pregnancy (baseline – 36 weeks, 19 scans). Total brain volume, gray matter volume, and cortical thickness were positively associated with one another, whereas lateral ventricles, CSF, and global QA demonstrated negative relationships with gray matter volume. Several summary metrics were not normally distributed—determined via Shapiro-Wilk's test for normality—so Spearman rank correlations were used. *Abbreviations:* QA = global anisotropy; GMV = gray matter volume; WMV = white matter volume; CSF = cerebrospinal fluid; Lat = lateral. Asterisks indicate significant correlation after FDR-correction ($q < 0.05$). ***$q < 0.001$; **$q < 0.01$, *$q < 0.05$, $^{+}q < 0.10$

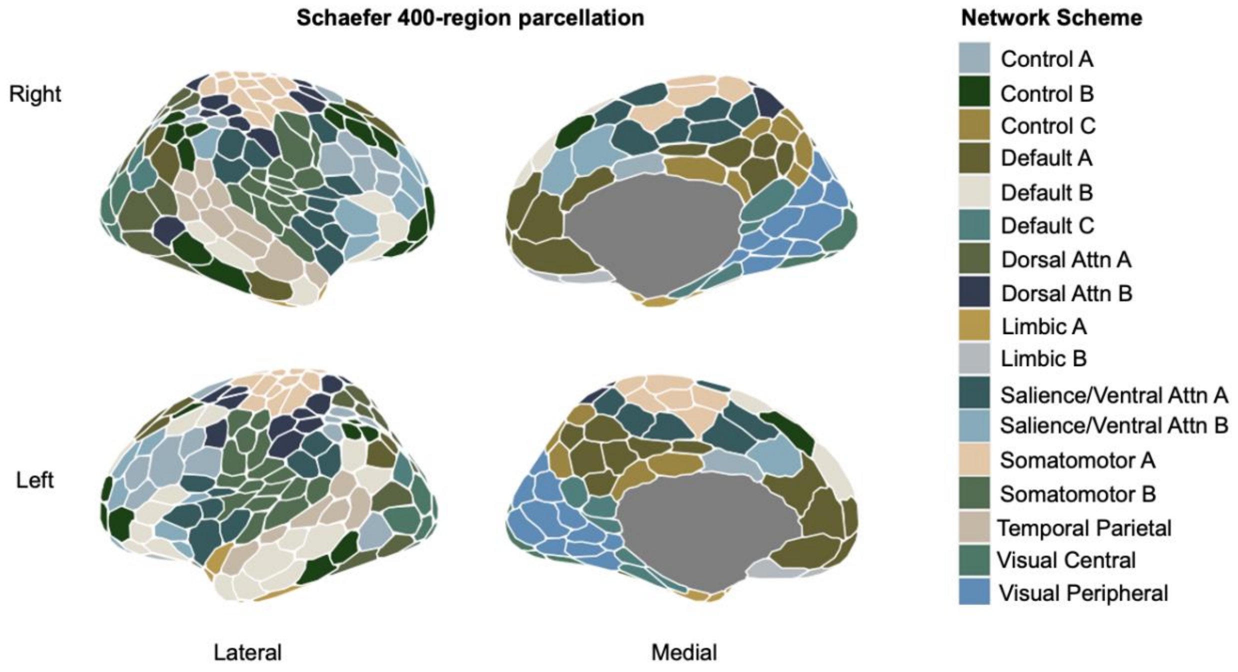

Supplementary Figure 2. The brain was parcellated into 400 cortical regions which were then assigned to one of eight networks based on previously identified anatomical and functional associations (Schaefer et al., 2018, Yeo et al., 2011). Colors indicate regional network membership.

Supplementary Table 1. Raw gray matter volume and correlations with gestation metrics by network

Network	Average	Range	Progesterone *N = 13*	17β-estradiol *N = 15*	Gestation week *N = 19*	Gestation week **controlling for total GMV*	Gestation week *FreeSurfer Results
Control A	0.64	0.62 – 0.67	-0.95***	-0.88***	-0.93***	-0.52*	-0.60**
Control B	0.67	0.66 – 0.69	-0.87***	-0.88***	-0.92***	-0.57*	-0.55*
Control C	0.68	0.65 - 0.70	-0.94***	-0.90***	-0.88***	-0.25	-0.78***
Default Mode A	0.70	0.69 – 0.72	-0.95***	-0.93***	-0.93***	-0.57*	-0.76***
Default Mode B	0.70	0.69 – 0.72	-0.95***	-0.87***	-0.91***	-0.29	-0.72***
Default Mode C	0.72	0.71 – 0.73	-0.86***	-0.72**	-0.69**	0.77***	-0.84***
Dorsal Attention A	0.66	0.64 – 0.68	-0.97***	-0.89***	-0.91***	-0.36	-0.64**
Dorsal Attention B	0.65	0.62 – 0.68	-0.97***	-0.91***	-0.95***	-0.72***	-0.55*
Salience Ventral Attn A	0.70	0.67 – 0.72	-0.97***	-0.91***	-0.95***	-0.71***	-0.92***
Salience Ventral Attn B	0.72	0.70 – 0.73	-0.92***	-0.89***	-0.92***	-0.41	-0.69**
Somatomotor A	0.65	0.61 – 0.67	-0.95***	-0.88***	-0.93***	-0.54*	-0.76***
Somatomotor B	0.70	0.67 – 0.73	-0.95***	-0.91***	-0.92***	-0.52*	-0.91***
Limbic A	0.76	0.74 – 0.79	-0.11	0.16	0.14	0.65**	-0.08
Limbic B	0.70	0.66 – 0.74	-0.48	-0.67**	-0.50*	0.27	-0.80***
Temporal Parietal	0.72	0.70 – 0.74	-0.97***	-0.92***	-0.89***	-0.10	-0.87***
Visual Central	0.67	0.65 – 0.69	-0.82***	-0.78***	-0.72***	0.51*	-0.66**
Visual Peripheral	0.70	0.69 – 0.72	-0.92***	-0.86***	-0.70**	0.71***	-0.72***

FDR-corrected at $q < 0.05$; *$q \leq 0.05$, **$q \leq 0.01$, ***$q \leq 0.001$

Supplementary Table 1. Raw gray matter volume (GMV) by network. Pearson's product-moment correlations were used to determine relationships between GMV and gestation week, estradiol, and progesterone (baseline – 36 weeks). The last two columns examine relationships between gestation week and average network GMV after controlling for global shifts in GMV (left) and deriving volumes via FreeSurfer in order to evaluate consistency across software pipelines (right).

Supplementary Table 2. Top gray matter volume ROIs associated with gestation week

Schaefer ROI	Network	Hemisphere	T-stat (raw)	T-stat (partial)
PFCd_1	ContA	Right	-6.36***	-5.33*
IPS_2	ContA	Left	-4.63***	-3.47*
PFCd_1	ContB	Left	-4.19***	-4.74*
PFCmp_1	ContB	Right	-8.38***	-3.72*
PFCmp_1	ContB	Left	-5.29***	-3.38*
pCun_3	ContC	Right	-10.10***	-3.26*
IPL_1	DMNA	Left	-10.94***	-4.23*
PFCd_3	DMNA	Left	-7.90***	-4.05*
pCunPCC_4	DMNA	Left	-10.02***	-3.67*
pCunPCC_2	DMNA	Right	-9.60***	-3.36*
PFCd_2	DMNA	Right	-8.55***	-3.33*
SPL_3	DorsAttnA	Left	-10.61***	-3.59*
PostC_6	DorsAttnB	Right	-13.84***	-5.35*
PostC_3	DorsAttnB	Left	-14.40***	-4.95*
PostC_7	DorsAttnB	Right	-7.80***	-4.56*
PostC_7	DorsAttnB	Left	-9.15***	-4.48*
PostC_9	DorsAttnB	Left	-8.63***	-4.08*
PostC_8	DorsAttnB	Left	-8.55***	-3.96*
FEF_2	DorsAttnB	Left	-8.12***	-3.77*
PostC_8	DorsAttnB	Right	-9.37***	-3.75*
PostC_1	DorsAttnB	Left	-12.00***	-3.53*
FEF_3	DorsAttnB	Left	-10.88***	-3.41*
Ins_1	SalVentAttnA	Left	-4.90***	-5.22*
FrOper_3	SalVentAttnA	Right	-11.57***	-4.26*
ParOper_3	SalVentAttnA	Left	-8.76***	-3.78*
FrMed_2	SalVentAttnA	Right	-4.21***	-3.37*
Ins_3	SalVentAttnB	Left	-7.09***	-5.07*
SomMotA_11	SomMotA	Left	-11.96***	-4.12*
SomMotA_12	SomMotA	Left	-4.90***	-4.11*
SomMotA_8	SomMotA	Left	-7.69***	-3.76*
SomMotA_5	SomMotA	Right	-10.67***	-3.23*
TempPar_3	TempPar	Left	-11.35***	-3.40*
PHC_2	DMNC	Right	-2.89*	4.09*
TempPole_3	LimbicA	Right	2.78	3.54*
ExStr_2	VisCent	Left	-4.71***	4.43*
ExStrInf_2	VisPeri	Right	0.18	4.57*

FDR-corrected at $q < 0.05$; *$q \leq 0.05$, **$q \leq 0.01$, ***$q \leq 0.001$

Supplementary Table 2. A subset of regions that demonstrate significant relationships between GMV and gestation week, before (raw) and after controlling for total GMV (partial) in a multivariate regression analysis. Negative 'partial' test-statistics suggest these regions-of-interest (ROIs) decrease at a rate greater than the global decrease; positive 'partial' test-statistics suggest these ROIs decrease at a rate slower than the global increase. Note, this is not an exhaustive list of results; see **Supplementary Data File 1** for complete reporting. *Abbreviations*: PostC = post central; FEF = frontal eye fields; Ins = insula; FrOper = frontal operculum; ParOper = parietal operculum; FrMed = frontal medial; ParOper = parietal operculum; IPL = inferior parietal lobule; PFCd = dorsal prefrontal cortex; pCunPCC = precuneus posterior cingulate cortex; PFCd = dorsal prefrontal cortex; PFCmd = medial posterior prefrontal cortex; IPS = intraparietal sulcus; SPL = superior parietal lobule.

Supplementary Table 3. Top Desikan-Killiany GMV ROIs associated with gestation week

DSK ROI	Hemisphere	T-stat (raw)	T-stat (partial)
Transverse Temporal	Bilateral	L: -8.59***, R: -5.05***	L: -4.70**, R: -3.80*
Lateral Orbital Frontal	Bilateral	L: -5.60***, R: -4.62***	L: -3.27*, R: -3.51*
Medial Orbital Frontal	Right	-6.03***	-4.80**
Lingual	Right	-4.98***	-3.58*
Posterior Cingulate	Left	-7.40***	-3.55*
Pericalcarine	Right	-2.78*	-3.44*
Precuneus	Right	-7.76***	-3.35*
Superior Temporal	Left	-7.54***	-3.26*
Insula	Right	-5.14***	-3.22*
Post Central Gyrus	Left	-7.36***	-3.04*
Pars Opercularis	Left	-7.30***	-3.00*
Superior Parietal	Left	-2.80*	2.94*
Inferior Parietal	Right	-0.70 (ns)	2.97*
Rostral Middle Frontal	Bilateral	L: -1.50 (ns), R: -2.48*	L: 3.84*, R: 3.23*
Supramarginal	Right	-1.04 (ns)	4.62**

FDR-corrected at $q < 0.05$; *$q \leq 0.05$, **$q \leq 0.01$, ***$q \leq 0.001$

Supplementary Table 3. Top GMV Desikan-Killiany regions of interest (ROIs) significantly associated with gestation week (raw), including those that remain significant after controlling for total GMV (partial). This allows us to identify regions with GMV decreasing at a rate greater (negative associations) or slower (positive associations) than the global decrease over the gestational window. Volumetric estimates were derived from FreeSurfer to cross-validate with a different pipeline and parcellation. N.b., this is not an exhaustive list; see full results reported in **Supplementary Data File 1**.

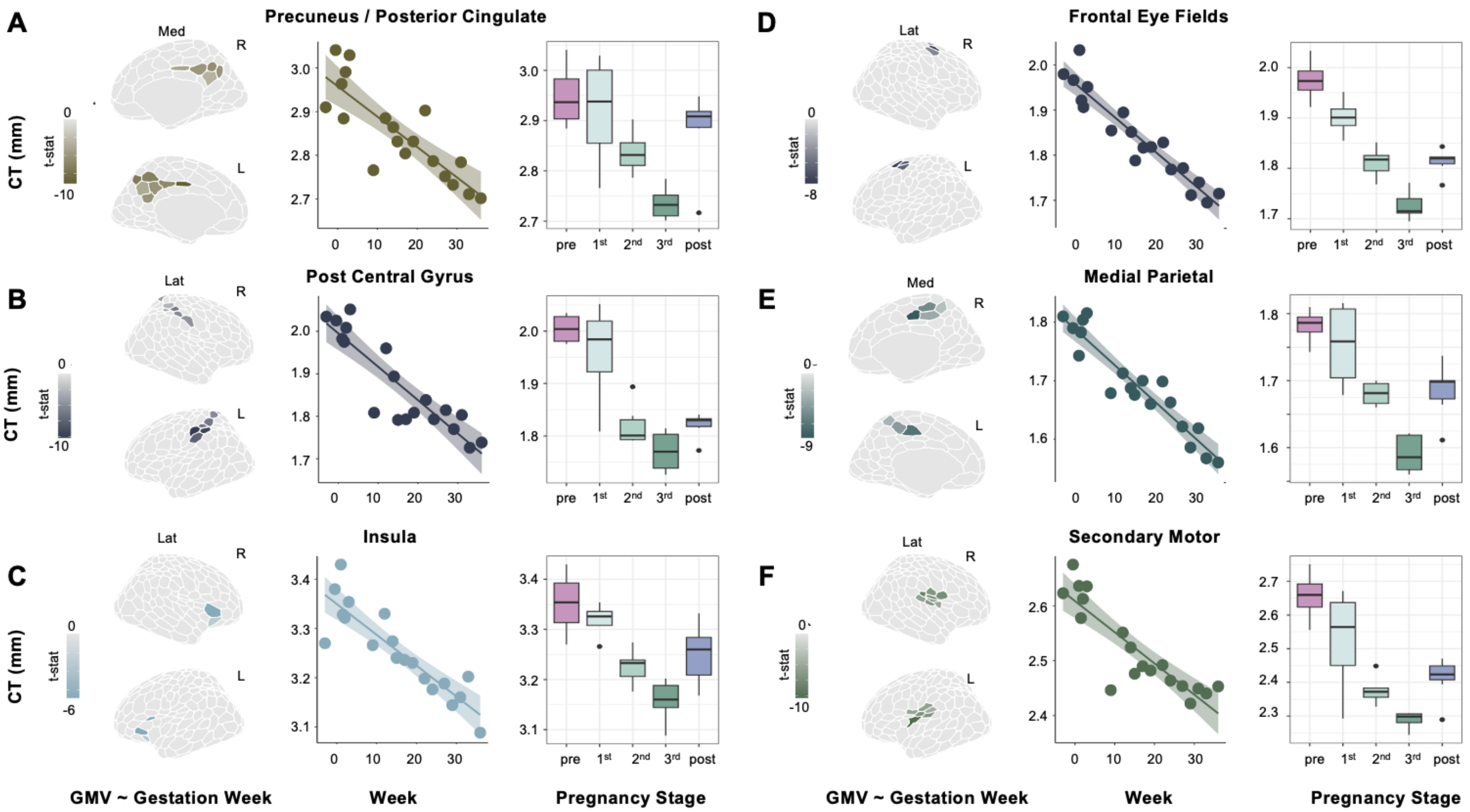

Supplementary Figure 3. Six representative regions, color coded by major subnetworks (see **Fig. S2**), that exhibit pronounced cortical thickness change across gestation. **A–F)** For each panel, we display results of a multivariate regression revealing significant associations between regional CT and gestation week (left; baseline – 36 weeks, 19 scans), Pearson's product-moment correlation between average CT of the regions-of-interest (ROIs) and gestation week (middle; baseline – 36 weeks, 19 scans), and summary ROI CT by pregnancy stage across the whole study (right; gestation and postpartum, 26 scans). Shaded regions in scatterplots represent a 95% confidence interval. For boxplots, each box represents the interquartile range (25th – 75th percentile) for each stage, with a horizontal line representing the median value (50th percentile). The whiskers indicate variability outside (±1.5) of this range. Outside values are >1.5 and <3 times the interquartile range beyond either end of the box. All statistical tests were corrected for multiple comparisons (FDR at $q < 0.05$). N.b., shown here are raw data values (see **Tables S4–5** and **Supplementary Data File 1** for exhaustive list). Brain visualizations created with R-package *ggseg* (Mowinckel and Vidal-Piñeiro, 2020).

Supplementary Table 4. Raw cortical thickness and associations with gestation metrics at network level

Network	Average	Range	Progesterone *N = 13*	17β-estradiol *N = 15*	Gestation week *N = 19*	Gestation Week *controlling for total CT*	Gestation Week *FreeSurfer Results*
Control A	1.87	1.75 – 2.02	-0.90***	-0.80***	-0.84***	-0.22	-0.59*
Control B	2.44	2.32 – 2.65	-0.89***	-0.85***	-0.83***	-0.05	-0.46
Control C	2.07	1.95 – 2.27	-0.90***	-0.80***	-0.75***	0.25	-0.83***
Default Mode A	2.69	2.57 – 2.91	-0.91***	-0.85***	-0.82***	-0.01	-0.83***
Default Mode B	2.57	2.46 – 2.78	-0.85***	-0.79***	-0.80***	0.27	-0.54*
Default Mode C	2.91	2.78– 3.05	-0.49*	-0.49	-0.40	0.86***	-0.81***
Dorsal Attention A	2.28	2.11 – 2.48	-0.91***	-0.82***	-0.77***	0.36	-0.46
Dorsal Attention B	1.92	1.77 – 2.12	-0.94***	-0.88***	-0.90***	-0.67*	-0.51
Salience Ventral Attn A	2.46	2.33 – 2.62	-0.95***	-0.91***	-0.91***	-0.79***	-0.93***
Salience Ventral Attn B	2.73	2.61 – 2.94	-0.91***	-0.86***	-0.86***	-0.39	-0.78**
Somatomotor A	1.76	1.52 – 2.23	-0.86***	-0.81***	-0.81***	-0.16	-0.62*
Somatomotor B	2.17	2.01 – 2.45	-0.93***	-0.90***	-0.89***	-0.77***	-0.83***
Limbic A	3.76	3.39 – 3.97	-0.39	-0.23	-0.32	0.62*	-0.46
Limbic B	2.81	2.53 – 3.12	-0.66*	-0.75**	-0.59**	0.26	-0.75**
Temporal Parietal	2.66	2.46 – 2.86	-0.94***	-0.91***	-0.80***	-0.03	-0.73**
Visual Central	2.24	1.89 – 2.50	-0.79**	-0.76**	-0.63**	0.46	-0.31
Visual Peripheral	2.34	2.10 – 2.50	-0.63*	-0.44	-0.22	0.81***	-0.56*

FDR-corrected at $q < 0.05$; *$q \leq 0.05$, **$q \leq 0.01$, ***$q \leq 0.001$

Supplementary Table 4. Raw cortical thickness (CT) by network. Pearson's product-moment correlations were used to determine relationships between cortical thickness and gestation week, estradiol, and progesterone (baseline – 36 weeks). The last two columns examine relationships between gestation week and average network CT after controlling for global shifts in CT (left) and deriving thickness estimates via FreeSurfer in order to evaluate consistency across software pipelines (right).

Supplementary Table 5. Top cortical thickness ROIs associated with gestation week

Schaefer ROI	Network	Hemisphere	T-stat (raw)	T-stat (partial)
PFCl_3	ContA	Left	-9.90***	-4.23*
PostC_2	DorsAttnB	Left	-10.20***	-4.94
PostC_3	DorsAttnB	Left	-9.50***	-4.05*
FEF_2	DorsAttnB	Left	-8.86***	-3.91*
FEF_3	DorsAttnB	Left	-7.80***	-3.90*
FEF_2	DorsAttnB	Right	-8.63***	-3.70*
ParMed_1	SalVentAttnA	Right	-11.57***	-5.72**
ParMed_1	SalVentAttnA	Left	-9.38***	-4.68*
Ins_2	SalVentAttnB	Left	-6.77***	-3.77*
Ins_1	SomB	Left	-11.41***	-5.22*
S2_1	SomB	Left	-10.39***	-4.72*
TempPole_4	LimbicA	Left	-1.48	4.73*
ExStrInf_5	VisPeri	Left	1.35	3.70*
StriCal_1	VisPeri	Right	4.51***	3.80*
ExStrInf_3	VisPeri	Left	1.39	3.98*
ExStrInf_2	VisPeri	Left	-0.21	4.17*
ExStrInf_4	VisPeri	Right	1.10	5.73**
Rsp_2	DMNC	Left	1.87	4.04*

FDR-corrected at $q < 0.05$; *$q \leq 0.05$, **$q \leq 0.01$, ***$q \leq 0.001$

Supplementary Table 5. A subset of regions that demonstrate significant relationships between cortical thickness and gestation week, before (raw) and after controlling for total thickness (partial) in a multivariate regression analysis. Note that cortical thickness in regions belonging to the Visual Peripheral, Limbic A, and Default Mode C Networks becomes positively associated with gestation week after controlling for global change. This suggests that these regions decline in thickness at a rate slower than the global decrease over the gestational window. Note, this is not an exhaustive list of results; see **Supplementary Data File 1** for complete reporting. *Abbreviations*: PostC = post central; FEF = frontal eye fields; ParMed = parietal medial; Ins = insula; S2 = Somatomotor 2; PFCl = lateral prefrontal cortex; ExStrInf = extra-striate inferior; StriCal = striate calcarine; TempPole = temporal pole; Rsp = retrosplenial.

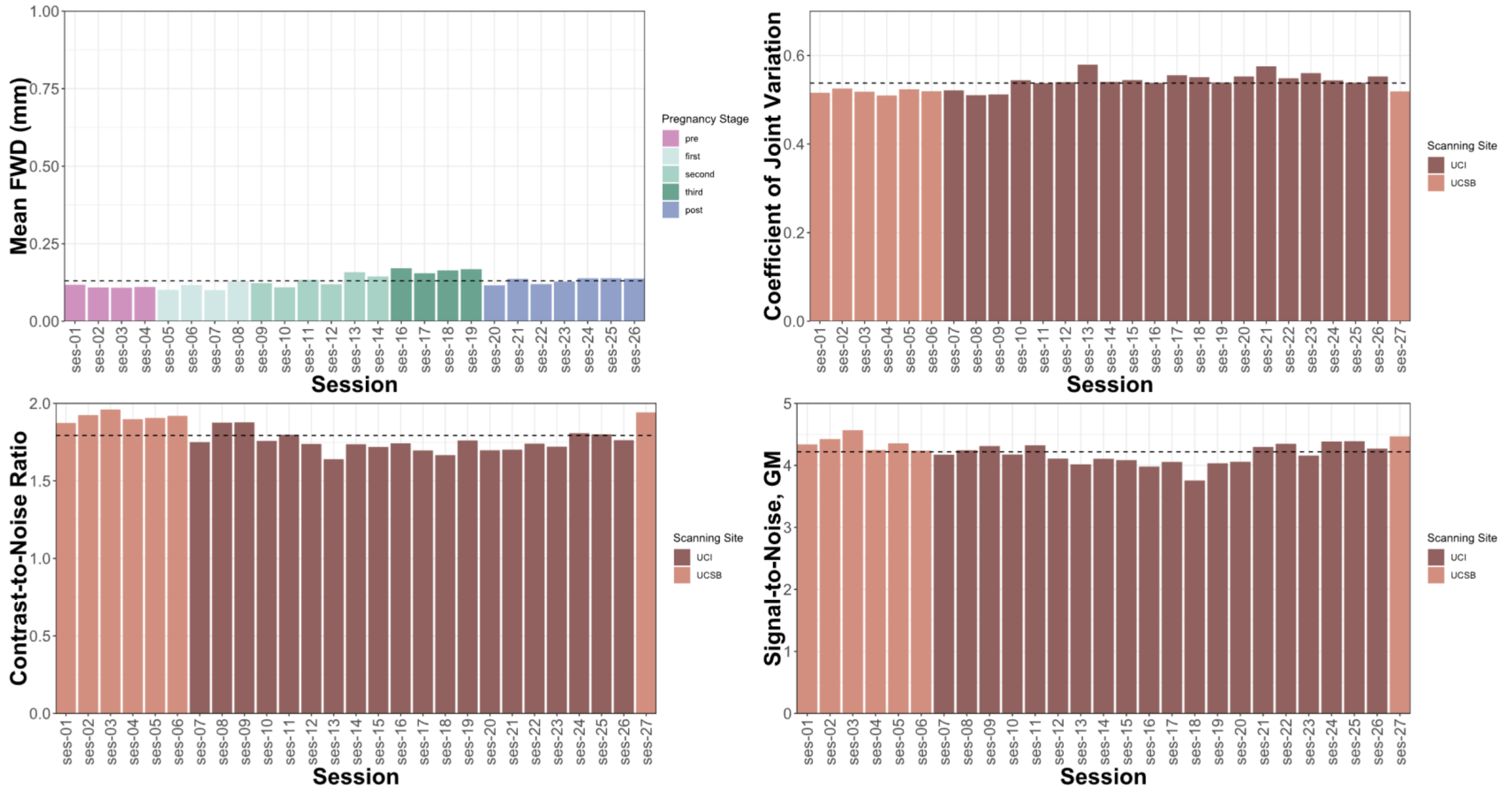

Supplementary Figure 4. Quality control estimates for this precision imaging experiment fell within standard ranges (see Esteban et al., 2017b); dashed horizontal line represents the average. **A)** Mean motion (i.e., framewise displacement) estimates were derived from N=17 available resting-state scans that followed each session's whole-brain T1 MPRAGE scan. Head motion was minimal compared to a conservative 'acceptable' limit of 1mm. **B–D)** Quality control estimates derived using the IQMs pipeline from *MRIQC*. **B)** Coefficient of Joint Variation is indicative of head motion and artifacts. **C)** Contrast-to-noise ratio evaluates separation of tissue distributions of GM and WM. **D)** Signal-to-noise ratio for gray matter. Cortical GMV reductions over pregnancy were still observable after controlling for these QC factors; however, the magnitude and location of these relationships were altered (see **Fig. S5**).

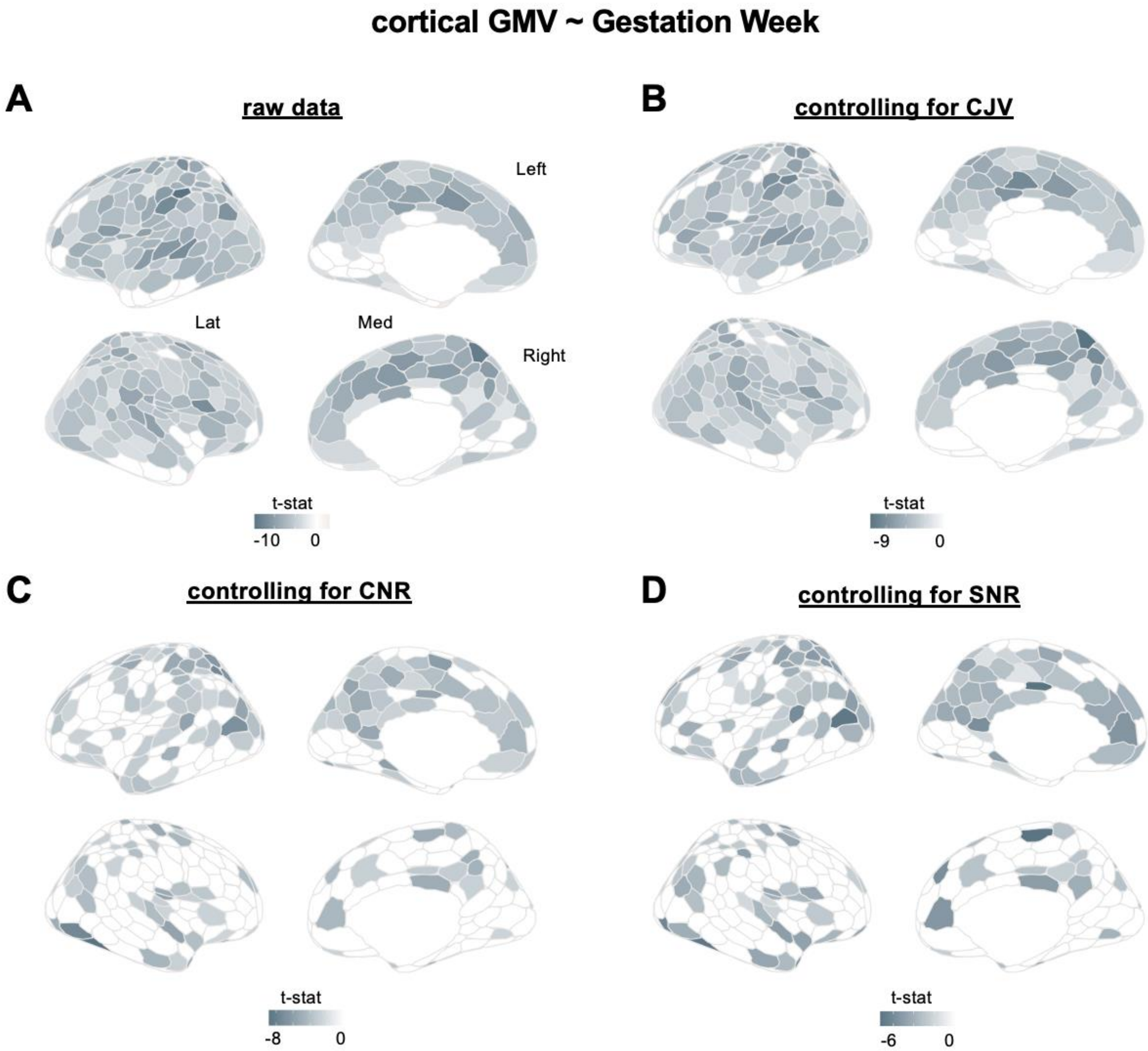

Supplementary Figure 5. A comparison of our raw (**A**) whole-brain GMV multivariate regression results and models (**B–C**) accounting for *MRIQC* quality control assessments (Esteban et al., 2017a). Controlling for these measures does not detract from our main finding suggesting cortical GMV reductions occur over gestation, especially within regions belonging to attention and somatosensory networks. However, there are observable decreases in CNR and SNR tied to gestation week, impacting the magnitude and location of our results. Given that data quality fell within the normal range, there may be meaningful reductions in signal to accompany volumetric reductions (e.g., regional increases in CSF paired with decreases in GMV) — a methodological nuance that warrants further exploration. *Abbreviations*: CJV = coefficient of joint variation; CNR = contrast-to-noise ratio; SNR = signal-to-noise ratio; Lat = lateral; Med = medial.

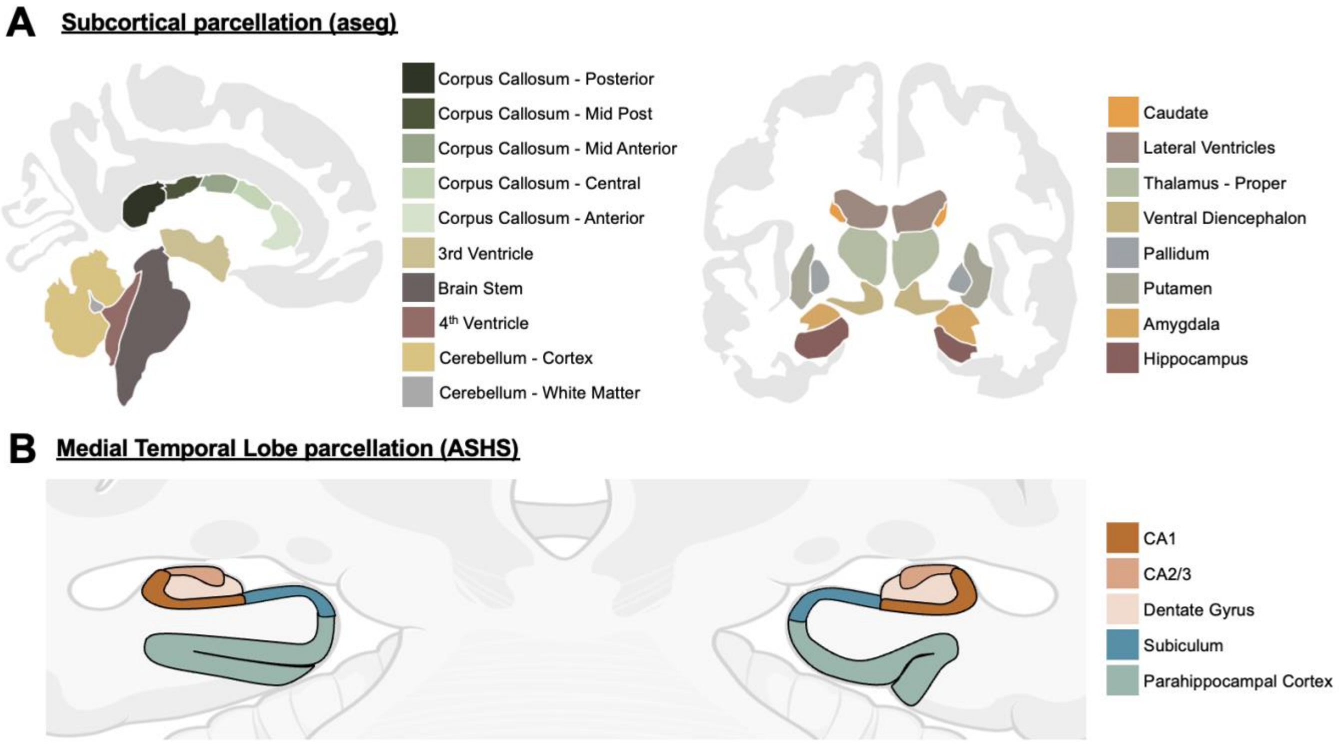

Supplementary Figure 6. Complete labelling of subcortical segmentations. **A)** Subcortical segmentation based upon the existence of an atlas ('aseg') containing probabilistic information on the location of structures via whole-brain T1w images. **B)** Cartoon depiction of medial temporal lobe segmentation determined via manual editing of the output of the Automatic Segmentation of Hippocampal Subfields (ASHS) software package. The participant's hippocampus and surrounding cortex were segmented into seven bilateral subregions. To note, perirhinal and entorhinal cortex subfields are not shown in this graphic due to slice coverage but are included in statistical analyses.

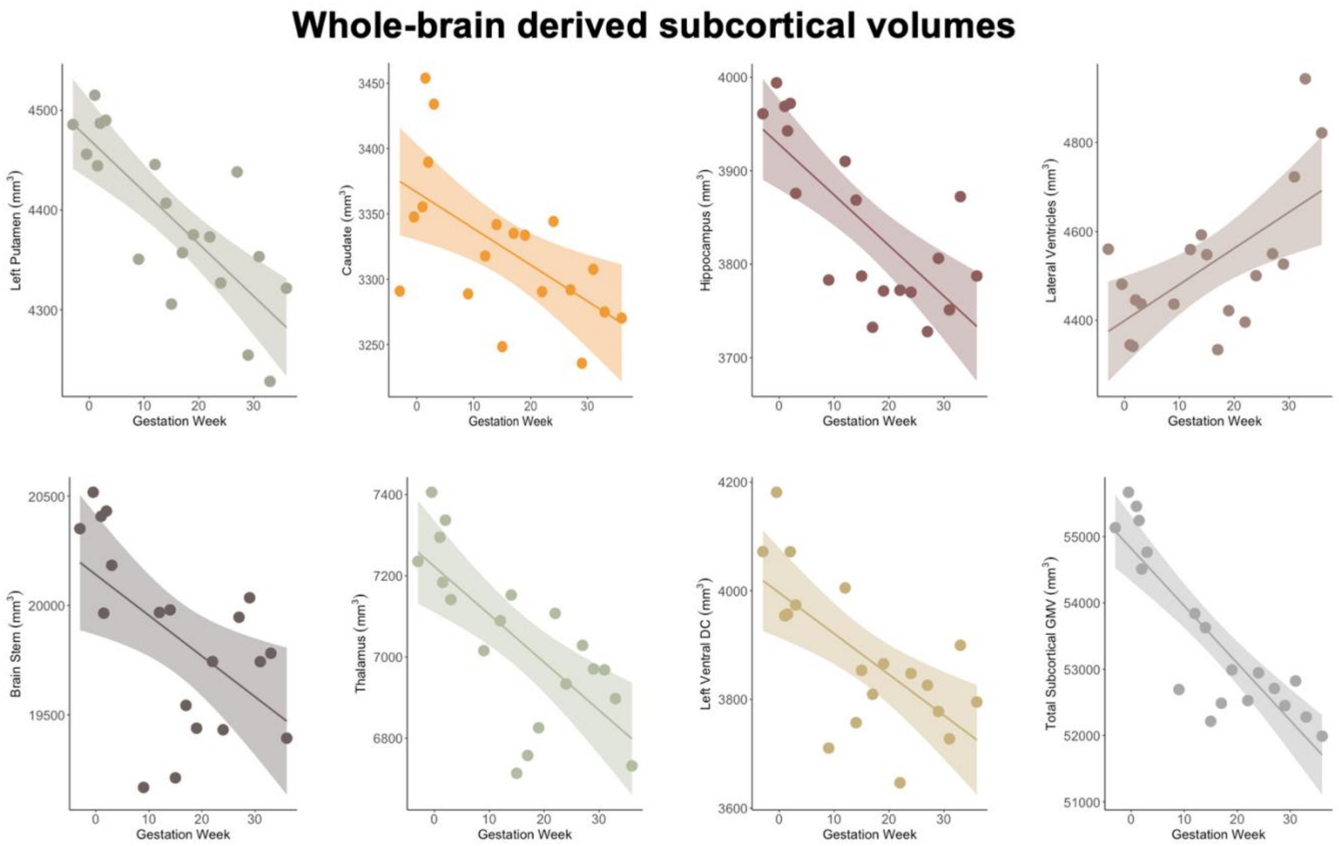

Supplementary Figure 7. Subcortical regions defined via the ‘aseg’ parcellation scheme (volume estimates derived via FreeSurfer) that demonstrate significant relationships with gestation week (baseline – 36 weeks; $q < .05$), as determined by linear regression models. Regions that displayed similar patterns across left and right hemispheres were averaged here; see Fig. 3A and Supplementary Table 6 for details. A significant linear model was fit for each region (shaded regions represent 95% confidence interval; solid line indicates model fit) and data were normally distributed. However, data patterns across a handful of structures suggest the potential for non-linear trends over gestation – a possibility that should be addressed in the future with larger cohorts.

Supplementary Table 6. Relationships between subcortical GMV and gestation week

aseg ROI	Hemisphere	T-stat
Ventral Diencephalon	Bilateral	L: -3.76**, R: -6.44***
Putamen	Left	-5.39***
Hippocampus	Bilateral	L: -3.35*, R: -5.10***
Thalamus	Bilateral	L: -3.31*, R: -3.03*
Lateral Ventricle	Bilateral	L: 3.20*, R: 3.40*
Brain Stem	--	-2.79*
Caudate	Bilateral	L: -2.60*, R: -2.68*

FDR-corrected at $q < 0.05$; *$q \leq 0.05$, **$q \leq 0.01$, ***$q \leq 0.001$

Supplementary Table 6. Significant relationships between subcortical regions of interest (derived from whole-brain T1w scan using Freesurfer, see *Methods*) and gestation week over the course of pregnancy (baseline – 36 weeks, 19 scans).

Supplementary Table 7. Medial temporal subregion volumes (mm^3) and associations with gestation week and hormones

	CA1	CA2/3	DG	Sub	ERC	PRC	PHC	Total Hipp Body
**Average (SD)**	**507.50 (25.45)**	**175.63 (18.60)**	497.72 (30.12)	265.33 (12.08)	735.02 (44.61)	2156.02 (67.35)	**1839.74 (76.00)**	1446.19 (74.51)
**Min**	**462.34**	**144.16**	451.15	244.29	643.16	2064.34	**1684.42**	1318.49
**Max**	**554.35**	**202.48**	561.98	281.68	836.64	2310.03	**1979.07**	1562.50
**Range**	**92.01**	**58.33**	110.83	37.38	193.48	245.69	**294.65**	244.01
**Gestation Week** adjusted R^2 *(q)*	**0.36[a]* (0.031)**	**0.41[a]* (0.027)**	-0.06 *n.s.*	-0.06 *n.s.*	0.03 *n.s.*	0.11 *n.s.*	**0.58* (0.0009)**	-0.06 *n.s.*
**Estradiol** adjusted R^2 *(q)*	0.10 *n.s.*	0.18 *n.s.*	-0.07 *n.s.*	0.03 *n.s.*	0.01 *n.s.*	0.06 *n.s.*	**0.60* (0.005)**	0.05 *n.s.*
**Progesterone** adjusted R^2 *(q)*	-0.02 *n.s.*	0.05 *n.s.*	-0.10 *n.s.*	-0.06 *n.s.*	-0.08 *n.s.*	0.08 *n.s.*	0.75 *n.s.*	-0.05 *n.s.*

FDR-corrected at $q < 0.05$ | n.s. indicates q > 0.05 | [a] denotes a quadratic relationship, otherwise linear | * remains significant with total volume correction

Supplementary Table 7. Medial temporal lobe subregion volumes in relation to gestation week and sex hormones. Regression models were used to determine associations between subregion volume and gestation week, estradiol, and progesterone (baseline – 36 weeks). Non-linear (CA1, CA2/3) and linear (PHC) volumetric decreases were observable across gestation (also see **Fig. 3B**). There was no significant change in other subregions or total volume of the hippocampal body (sum of CA1, CA2/3, DG, and Sub), or in the parahippocampal gyrus (**Fig. S8**). *Abbreviations*: DG = dentate gyrus; Sub = subiculum; ERC = entorhinal cortex; PRC = perirhinal cortex; PHC = parahippocampal cortex.

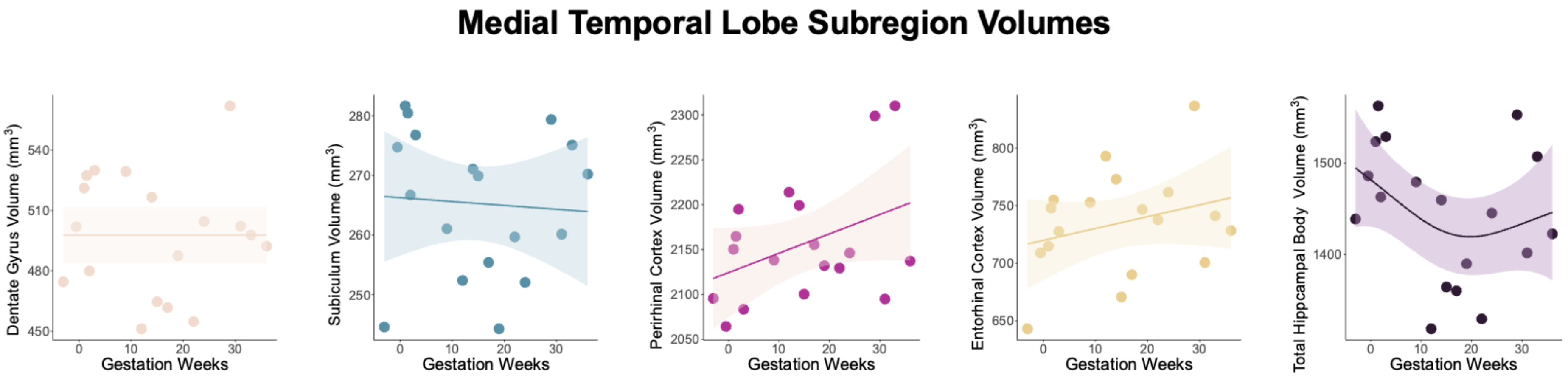

Supplementary Figure 8. Medial temporal subregion volumes across gestation. Linear regression models reveal no significant relationships ($p > 0.05$). Shaded regions represent 95% confidence interval. However, data patterns across a handful of structures suggest the potential for non-linear trends over gestation – a possibility that should be addressed in the future with larger cohorts.

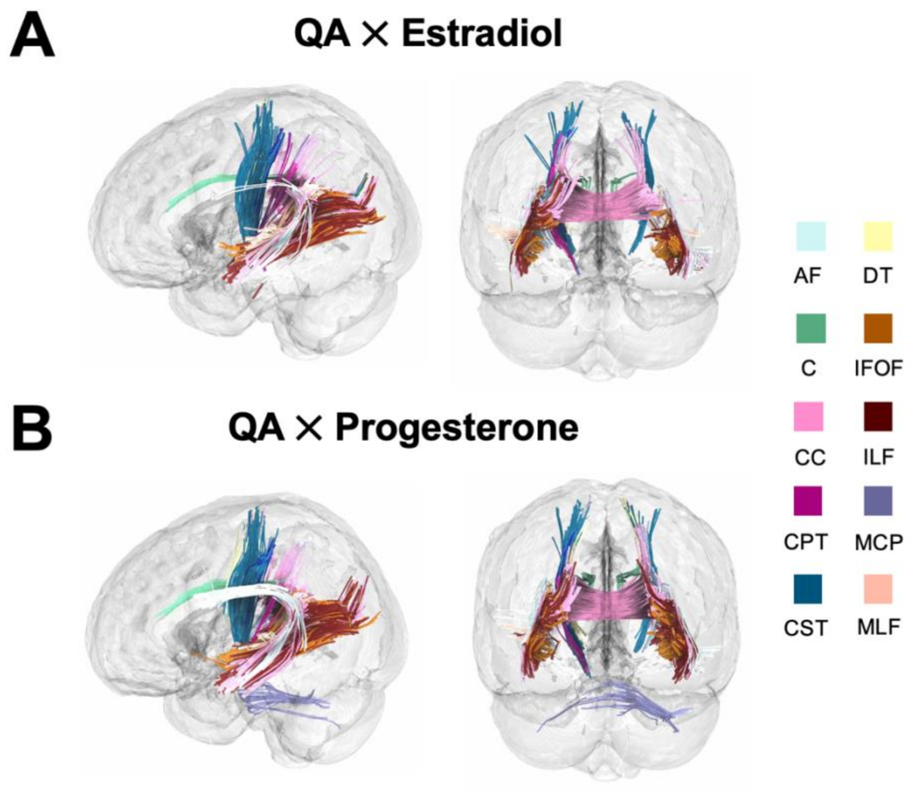

Supplementary Figure 9. Relationships between white matter integrity and hormones throughout gestation (baseline – 36 weeks). A) White matter tracts in which quantitative anisotropy (QA) was significantly positively associated with estradiol (FDR $q < .001$). B) White matter tracts in which QA was significantly positively associated with progesterone (FDR $q < .001$). See Supplementary Table 9 for complete list of tracts with a significant correlation between QA and estradiol/progesterone. *Abbreviations:* AF = arcuate fasciculus; C = cingulum bundle; CC = corpus callosum; CPT = corticopontine tracts; CST = corticospinal tracts; DT = dentatorubrothalamic tract; IFOF = inferior frontal occipital fasciculus; ILF = inferior longitudinal fasciculus; MCP = middle cerebellar peduncle; MLF = middle longitudinal fasciculus.

Supplementary Table 9. Full report of quantitative anisotropy results by tract that show positive associations with gestation week and sex hormones

Region of Interest	Number of tracts			Mean length (mm)			Diameter (mm)			Volume (mm^3)		
	Gest Week	*Progesterone*	*Estradiol*	*Gest Week*	*Progesterone*	*Estradiol*	*Gest Week*	*Progesterone*	*Estradiol*	*Gest Week*	*Progesterone*	*Estradiol*
Anterior_Commissure	19	11	–	55.3	55.8	–	3.9	3.3	–	667	482	–
Arcuate_Fasciculus_L	111	88	88	56.1	54.8	56.3	5.9	6.7	5.7	1548	1940	1444
Arcuate_Fasciculus_R	470	129	191	62.7	59.2	60.2	7.7	6.5	6.2	2942	1959	1814
Cerebellum_L	28	–	–	53.2	–	–	3.8	–	–	600	–	–
Cingulum_Frontal_Parahippocampal_L	22	–	–	52.5	–	–	3.1	–	–	402	–	–
Cingulum_Frontal_Parietal_L	74	54	11	54.1	54.7	52.0	5.3	4.2	3.1	1174	753	382
Cingulum_Frontal_Parietal_R	193	33	43	57.2	51.9	53.5	6.5	4.6	3.5	1896	847	502
Cingulum_Parolfactory_L	37	–	–	59.6	–	–	3.2	–	–	478	–	–
Cingulum_Parolfactory_R	66	13	21	58.0	51.8	56.4	3.2	1.9	1.8	478	140	146
Corpus_Callosum_Body	1126	192	166	63.0	58.3	57.5	14.2	8.9	8.4	10007	3604	3216
Corpus_Callosum_Forceps_Major	1935	765	631	65.7	60.9	61.8	13.8	10.9	9.5	9815	5677	4427
Corpus_Callosum_Forceps_Minor	139	–	–	57.0	–	–	7.3	–	–	2355	–	–
Corpus_Callosum_Tapetum	1988	639	599	59.6	55.7	55.8	17.4	14.2	13.0	14265	8877	7394
Corticopontine_Tract_Occipital_L	54	–	–	54.8	–	–	3.8	–	–	611	–	–
Corticopontine_Tract_Occipital_R	45	–	–	54.8	–	–	3.1	–	–	426	–	–
Corticopontine_Tract_Parietal_L	59	16	32	55.3	52.8	54.0	4.9	3.0	4.2	1059	367	754
Corticopontine_Tract_Parietal_R	129	–	–	57.6	–	–	6.5	–	–	1936	–	–
Corticospinal_Tract_L	385	372	216	61.4	61.6	61.4	10.0	10.3	8.8	4833	5083	3775
Corticospinal_Tract_R	200	84	49	59.9	58.6	60.5	7.0	6.2	5.2	2314	1775	1295
Corticostriatal_Tract_Posterior_L	154	31	46	55.2	54.7	55.8	6.3	4.4	4.9	1705	836	1072
Corticostriatal_Tract_Posterior_R	115	18	–	54.7	53.0	–	7.1	4.3	–	2145	779	–
Corticostriatal_Tract_Superior_L	39	10	–	54.7	54.0	–	4.8	3.3	–	1005	448	–
Dentatorubrothalamic_Tract_L	77	66	38	57.8	59.3	56.6	6.0	6.5	4.9	1626	1961	1069
Dentatorubrothalamic_Tract_R	16	28	–	55.4	57.0	–	2.7	4.2	–	328	800	–
Fornix_R	13	–	–	52.5	–	–	2.2	–	–	197	–	–
Inferior_Fronto_Occipital_Fasciculus_L	284	166	248	58.0	57.1	57.0	8.6	8.3	7.7	3332	3109	2683
Inferior_Fronto_Occipital_Fasciculus_R	1132	218	263	59.3	58.6	56.5	10.4	8.2	7.1	5042	3132	2265
Inferior_Longitudinal_Fasciculus_L	1019	498	553	64.3	62.4	63.2	10.6	9.9	9.5	5703	4804	4511
Inferior_Longitudinal_Fasciculus_R	1122	497	360	61.4	58.8	58.3	11.6	10.0	7.7	6481	4588	2692
Medial_Lemniscus_L	19	20	15	55.3	56.2	54.8	4.1	4.1	3.3	717	724	479
Medial_Lemniscus_R	55	–	–	57.5	–	–	5.3	–	–	1265	–	–
Middle_Cerebellar_Peduncle	11	53	–	52.9	59.1	–	3.3	6.3	–	449	1862	–
Middle_Longitudinal_Fasciculus_L	191	35	125	53.6	52.1	53.0	6.3	4.3	6.2	1686	772	1578
Middle_Longitudinal_Fasciculus_R	176	–	10	54.2	–	51.2	6.6	–	2.3	1862	–	211
Parietal_Aslant_Tract_R	75	–	20	53.7	–	53.0	4.2	–	3.2	734	–	415
Superior_Longitudinal_Fasciculus1_R	34	–	–	54.9	–	–	3.9	–	–	640	–	–
Thalamic_Radiation_Posterior_L	35	–	15	54.8	–	56.7	4.3	–	2.8	794	–	340
Thalamic_Radiation_Posterior_R	35	–	–	54.7	–	–	4.2	–	–	748	–	–

Supplementary Table 9. Tract statistics for QA among tracks that were identified (via correlational tractography analysis) as being significantly positively associated with gestation week (FDR $q < 0.001$), progesterone (FDR $q < 0.001$), and estradiol (FDR $q = 0.001$). Tracts are labeled by hemisphere (L, left; R, right) and report the number of tracts, mean length, diameter, and volume. Dashes signify no significant relationships between the pregnancy outcome variable and respective tract, as determined by the FDR value determined in DSI studio.

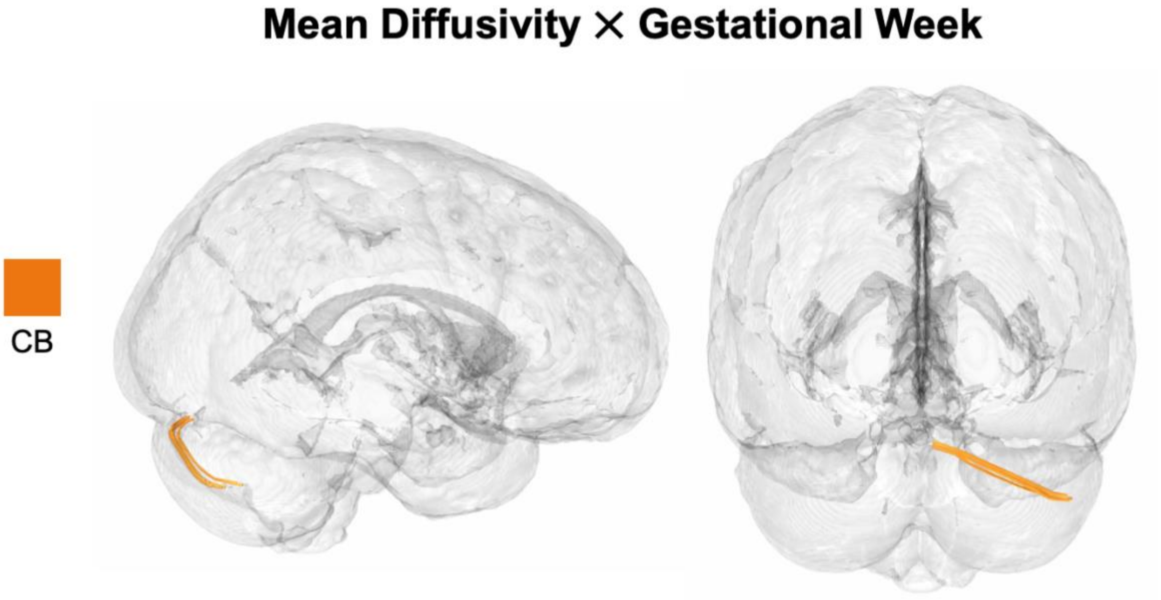

Supplementary Figure 10. Relationships between white matter integrity and gestation (baseline – 36 weeks). White matter tracts in which mean diffusivity (MD) was positively associated with gestational week (FDR $q < .02$). Correlations were positive, but because lower MD values typically indicate worse microstructural integrity these findings indicate tracts where microstructural integrity declined during gestation. *Abbreviations:* CB = cerebellum

Supplementary Table 10. Full report of mean diffusivity results by tract that show positive associations with gestation week and estradiol

	Number of Tracts			Mean Length			Diameter (mm)			Volume (mm^3)		
Region of Interest	Gest Week	Progesterone	Estradiol	Gest Week	Progesterone	Estradiol	Gest Week	Progesterone	Estradiol	Gest Week	Progesterone	Estradiol
Cerebellum_L	-	184	20	-	54.2	53.8	-	6.6	3.3	-	1859	455
Cerebellum_R	12	65	16	51.8	52.7	53.0	3.0	3.9	3.3	364	643	466
Inferior_Fronto_Occipital_Fasciculus_L	-	-	15	-	-	54.2	-	-	2.6	-	-	281

Supplementary Table 10. Mean diffusivity tract statistics for those significantly correlated to gestational week (FDR $q < 0.02$) and progesterone (FDR $q = 0.01$). DSI Studio recommends reporting tracts that with a significance level of FDR < 0.2. Based on this criteria, we have included tracts with MD associated with estradiol (FDR $q = 0.06$) for completeness. Regions are labeled by hemisphere (L, left; R, right) and reported on by number of tracts, mean length, diameter, and volume. Dashes indicate no statistical relationship. Higher MD values typically indicate worse microstructural integrity.

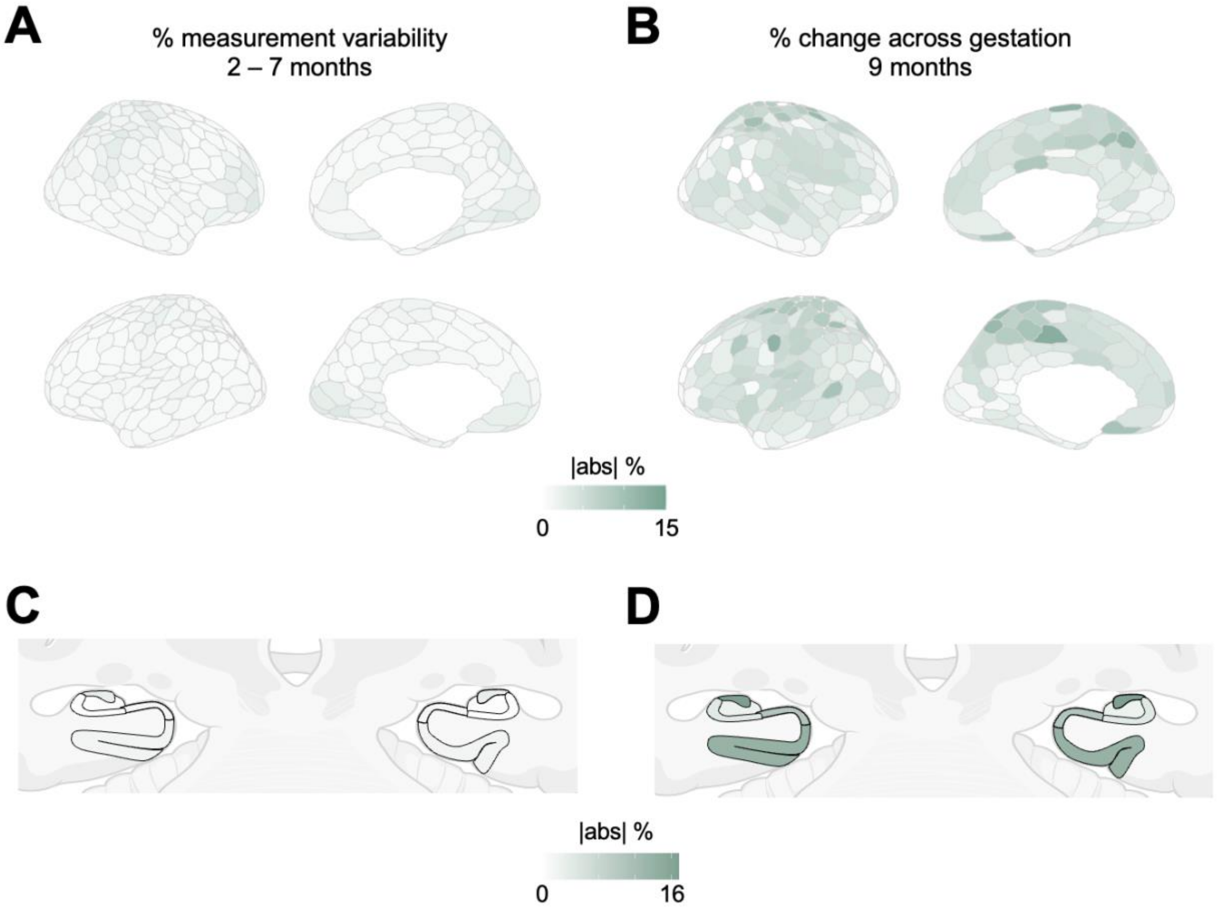

Supplementary Figure 11. We further contextualized our results by utilizing the Day2Day dataset from Filevich et al., 2017, wherein eight individuals (6F/2M) underwent brain imaging over an extended longitudinal window (2–7 months). Whole-brain T1w images from each subject were run through the same protocols used in our own data, ensuring proper comparison of the output from both datasets. **A)** Using Day2Day T1w data, we computed the degree of measurement variability (see *Methods*) for each region of the brain, allowing us to derive an estimate of typical day-to-day variability and measurement error. **B)** The change that occurs during pregnancy (baseline vs. 36 weeks gestation) goes far beyond what can be deemed 'typical' variability in the brain over a similar time course. A similar comparison was made for medial temporal lobe volumes which **D)** exhibit changes during pregnancy that exceed **C)** normative variability. Note: *Brain visualizations created with R-package ggseg (A-B; Mowinckel and Vidal-Piñeiro, 2020) and BioRender.com (C-D).*

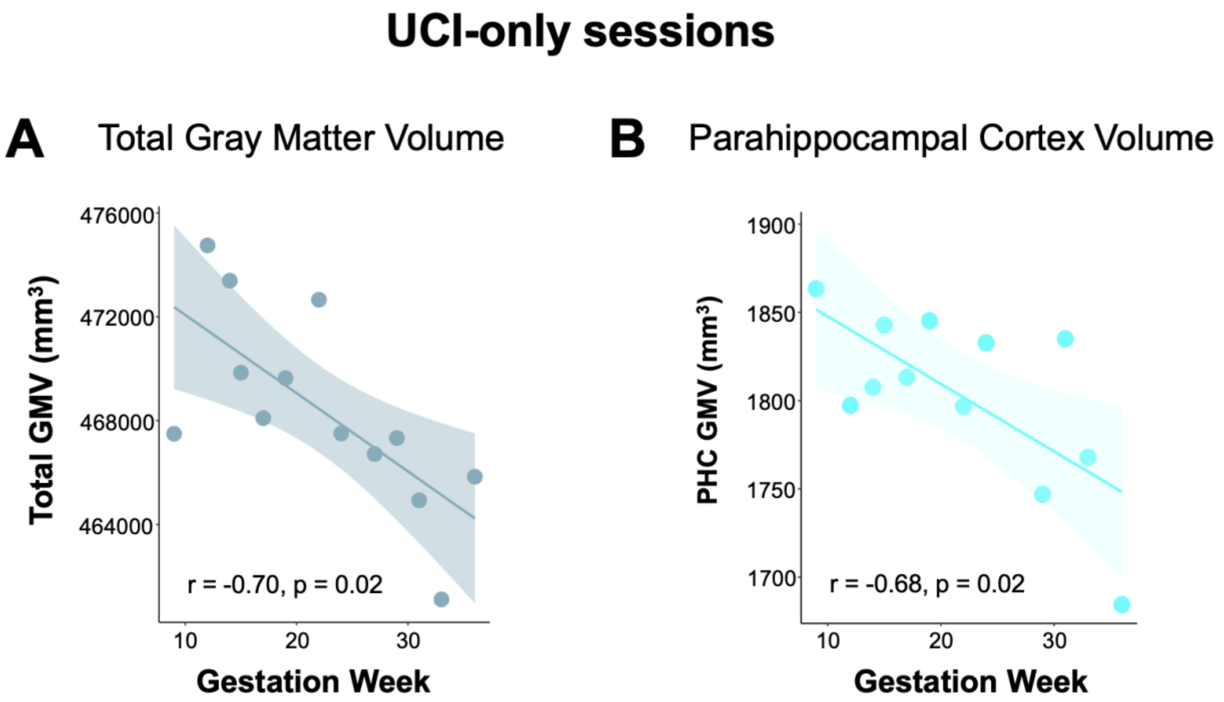

Supplementary Figure 12. Representative relationships between neuroanatomy and gestation when evaluating UCI sessions only (n=13). A) Gestation week remains significantly tied to decreasing total GMV (Pearson's product-moment correlation; two-sided test). Shaded regions represent 95% confidence interval. Additionally, most large-scale brain networks remain negatively associated with GMV across gestation, except for Visual (Central, Peripheral), Limbic (A,B), and Default Mode Network (C) (see Table S1 for patterns across all sessions). B) Similarly, gestation week remains significantly associated with decreasing parahippocampal cortex volume.
