# Supplementary material for: Neuroanatomical changes observed over the course of a human pregnancy
Source: Nat Neurosci. 2024 Sep 16;27(11):2253–60. doi: 10.1038/s41593-024-01741-0 (PMC11537970; doi:10.1038/s41593-024-01741-0)
Supplement: Supplementary file 2 — Reporting Summary [file 41593_2024_1741_MOESM2_ESM.pdf]

nature portfolio

Corresponding author(s): Laura Pritschet, Elizabeth R. Chrastil, & Emily G. Jacobs

Last updated by author(s): 06/30/2024

Reporting Summary

Nature Portfolio wishes to improve the reproducibility of the work that we publish. This form provides structure for consistency and transparency in reporting. For further information on Nature Portfolio policies, see our Editorial Policies and the Editorial Policy Checklist.

Statistics

For all statistical analyses, confirm that the following items are present in the figure legend, table legend, main text, or Methods section.

n/a	Confirmed	
☐	☒	The exact sample size (n) for each experimental group/condition, given as a discrete number and unit of measurement
☐	☒	A statement on whether measurements were taken from distinct samples or whether the same sample was measured repeatedly
☐	☒	The statistical test(s) used AND whether they are one- or two-sided<br>*Only common tests should be described solely by name; describe more complex techniques in the Methods section.*
☐	☒	A description of all covariates tested
☐	☒	A description of any assumptions or corrections, such as tests of normality and adjustment for multiple comparisons
☐	☒	A full description of the statistical parameters including central tendency (e.g. means) or other basic estimates (e.g. regression coefficient) AND variation (e.g. standard deviation) or associated estimates of uncertainty (e.g. confidence intervals)
☐	☒	For null hypothesis testing, the test statistic (e.g. F, t, r) with confidence intervals, effect sizes, degrees of freedom and P value noted<br>*Give P values as exact values whenever suitable.*
☒	☐	For Bayesian analysis, information on the choice of priors and Markov chain Monte Carlo settings
☒	☐	For hierarchical and complex designs, identification of the appropriate level for tests and full reporting of outcomes
☐	☒	Estimates of effect sizes (e.g. Cohen's d, Pearson's r), indicating how they were calculated

Our web collection on statistics for biologists contains articles on many of the points above.

Software and code

Policy information about availability of computer code

Data collection	All data collection was done on a Siemens 3T Prisma with software version MR E11. All sequences were standard Siemens protocols, with the exception to the T2-hippocampal scan: A T2-weighted (T2w) turbo spin echo (TSE) scan was also acquired with an oblique coronal orientation positioned orthogonally to the main axis of the hippocampus (TR/TE = 9860/50 ms, flip angle = 122°, 0.4 × 0.4 mm2 in-plane resolution, 2 mm slice thickness, 38 interleaved slices with no gap, total acquisition time = 5:42 min). No other custom software was used for data collection.
Data analysis	The following software packages were used:<br>Advanced Normalization Tools (ANTs), version 2.1.0<br>FreeSurfer, version 7<br>Automatic Segmentation of Hippocampal Subfields (ASHS), version 7/2018<br>IQM Pipeline from MRIQC, version 23.1<br>Matlab, version 2022a<br>QSIprep, version 0.15.3<br>DSI Studio, version Chen-2022-07-31<br>R/R Studio, version 3.4.4<br>ITK-SNAP, v.3.8.0-b

For manuscripts utilizing custom algorithms or software that are central to the research but not yet described in published literature, software must be made available to editors and reviewers. We strongly encourage code deposition in a community repository (e.g. GitHub). See the Nature Portfolio guidelines for submitting code & software for further information.

Data

Policy information about availability of data

All manuscripts must include a data availability statement. This statement should provide the following information, where applicable:

- Accession codes, unique identifiers, or web links for publicly available datasets
- A description of any restrictions on data availability
- For clinical datasets or third party data, please ensure that the statement adheres to our policy

The dataset consists of 26 MRI scans (T1w, T2w, and diffusion scans) alongside state-dependent measures and serum assessments of ovarian sex hormones for each session. The data is publicly available on https://openneuro.org/datasets/ds005299.

Research involving human participants, their data, or biological material

Policy information about studies with human participants or human data. See also policy information about sex, gender (identity/presentation), and sexual orientation and race, ethnicity and racism.

Reporting on sex and gender	Our study focused on a single female participant to explore how pregnancy shapes the human brain.
Reporting on race, ethnicity, or other socially relevant groupings	The subject was white.
Population characteristics	This was a precision imaging study of one 38-year old primiparous woman.
Recruitment	Our participant (corresponding author E.R.C.) was a healthy primiparous woman who underwent in-vitro fertilization (IVF) to achieve pregnancy. The project was conceived by E.R.C. and she wished to use herself as the participant, as has been done in previous "dense-sampling" studies (cf. Poldrack et al., 2015; Pritschet et al., 2020).
Ethics oversight	The participant gave written informed consent and the study was approved by the University of California, Irvine Human Subjects Committee.

Note that full information on the approval of the study protocol must also be provided in the manuscript.

Field-specific reporting

Please select the one below that is the best fit for your research. If you are not sure, read the appropriate sections before making your selection.

☒ Life sciences ☐ Behavioural & social sciences ☐ Ecological, evolutionary & environmental sciences

For a reference copy of the document with all sections, see nature.com/documents/nr-reporting-summary-flat.pdf

Life sciences study design

All studies must disclose on these points even when the disclosure is negative.

Sample size	We used precision imaging to deeply-phenotype, densely-sample an individual over the gestational window. As this study was the first of it's kind, our sample size was an N=1 design. Although this limits the generalizability of our findings, this project serves as a proof-of-concept, showcasing the value and feasibility of studying a woman's brain during the transition to motherhood.
Data exclusions	no history of neuropsychiatric diagnosis, endocrine disorders, prior head trauma or history of smoking
Replication	This is the first study of it's kind; therefore, there are no study replications as of yet. However, to reproduce our results internally across software packages, we also ran the T1w data through the longitudinal FreeSurfer cortical thickness pipeline (Dale et al., 1999), which corroborated our finding that gray matter volume declines throughout gestation (e.g., successful internal replication). This pattern of results not only held across software packages, but also brain parcellations (e.g., Schaefer 400-cortical atlas and Desikan-Killiany cortical atlas).
Randomization	This was an observational study design, and therefore not randomized.
Blinding	For medial temporal lobe segmentation, scans were randomized and segmentation was performed in a random order, blind to pregnancy stage. No other blinding was applicable, given the observational study of brain changes in response to advancing gestational week.

Reporting for specific materials, systems and methods

We require information from authors about some types of materials, experimental systems and methods used in many studies. Here, indicate whether each material, system or method listed is relevant to your study. If you are not sure if a list item applies to your research, read the appropriate section before selecting a response.

Materials & experimental systems

n/a	Involved in the study
☒	☐ Antibodies
☒	☐ Eukaryotic cell lines
☒	☐ Palaeontology and archaeology
☒	☐ Animals and other organisms
☒	☐ Clinical data
☒	☐ Dual use research of concern
☒	☐ Plants

Methods

n/a	Involved in the study
☒	☐ ChIP-seq
☒	☐ Flow cytometry
☐	☒ MRI-based neuroimaging

Magnetic resonance imaging

Experimental design

Design type: Structural & Diffusion MRI

Design specifications: No task-based fMRI used in this manuscript.

Behavioral performance measures: N/A; no performance metrics collected

Acquisition

Imaging type(s): Structural

Field strength: 3

Sequence & imaging parameters: High-resolution anatomical scans were acquired using a T1-weighted (T1w) magnetization prepared rapid gradient echo (MPRAGE) sequence (TR = 2500 ms, TE = 2.31 ms, T1 = 934 ms, flip angle = 7°, 0.8 mm thickness) followed by a gradient echo fieldmap (TR = 758 ms; TE1 = 4.92 ms; TE2 = 7.38 ms; flip angle = 60°). A T2-weighted (T2w) turbo spin echo (TSE) scan was also acquired with an oblique coronal orientation positioned orthogonally to the main axis of the hippocampus (TR/TE = 9860/50 ms, flip angle = 122°, 0.4 × 0.4 mm2 in-plane resolution, 2 mm slice thickness, 38 interleaved slices with no gap, total acquisition time = 5:42 min).

Area of acquisition: T1-weighted and dMRI scans = whole-brain
T2-weighted scan = high-resolution imaging of medial temporal lobe

Diffusion MRI ☒ Used ☐ Not used

Parameters: TR = 4300 ms, echo time = 100.2 ms, 139 directions, b-max = 4990, FoV = 259 x 259 mm, 78 slices, 1.7986 x 1.7986 x 1.8 mm voxel resolution

Preprocessing

Preprocessing software:

Gray Matter Volume & Cortical Thickness:
Advanced Normalization Tools (ANTs), version 2.1.0
FreeSurfer, version 7

T2-weighted MTL scans:
Automatic Segmentation of Hippocampal Subfields (ASHS), version 7/2018

Diffusion imaging:
QSIprep, version 0.15.3
DSI Studio, version Chen-2022-07-31

Normalization:

Normalization differed by modality due to inherent limitations of applicable processing pipelines.

Gray Matter Volume & Cortical Thickness:
All analyses were kept in native subject-space to limit the amount of warping and leverage the advantages of a precision imaging design.

T2-weighted MTL scans:
T2w images were registered to the segmentation template (see below) using ANTs deformable registration.

Diffusion imaging:
Initial preprocessing through QSIprep normalized diffusion images to the skull-stripped T1w images. Diffusion images were then reconstructed in MNI space using DSI studio’s Q-space Diffeomorphic Reconstruction.

Normalization template

T2-weighted MTL scans:
Princeton Young Adult 3T ASHS Atlas Template (n=24, mean age = 22.5; Aly & Turk-Browne, 2016).

Diffusion imaging:
All diffusion images were reconstructed using the ICBM152 template.

Noise and artifact removal

Gray Matter Volume & Cortical Thickness:
All T1-weighted images underwent denoising ('denoiseImage') and N4 bias field correction ('N4BiasFieldCorrection') for field inhomogeneity via ANTs.

T2-weighted MTL scans:
All T2-weighted MTL images underwent denoising ('denoiseImage') via ANTs.

Diffusion:
All diffusion images underwent denoising, motion and distortion correction using MRtrix3's dwidenoise and dwibiascorrect with the N4 algorithm. All diffusion images were quality checked using DSI studio's `QC1: SRC Files Quality Control. All images passed QC checks.

Motion:
Mean framewise displacement (FWD) estimates from gestation sessions with a 10-minute resting state scan (n = 18) were used to indirectly assess whether motion increased throughout pregnancy. Average FWD (millimeters) was extremely minimal across the entire experiment (M = 0.13, SD = 0.02, range = 0.09–0.17) and varied only slightly by pregnancy stage (pre: M = 0.11, SD = 0.004; first: M = 0.11, SD = 0.01; second: M = 0.13, SD = 0.02; third: M = 0.16, SD = 0.007; post: M = 0.13, SD = 0.01). While mean FWD did correspond with gestation week (r = 0.88, p < .001), controlling for this did not alter our main findings (e.g., total GMV negatively associated with gestation; partial correlation: r = -0.87, p < 0.001) owing to the fact that motion differences between stages were minuscule.

Volume censoring

Gray Matter Volume & Cortical Thickness:
All images were visually assessed for QC. Further, we computed quality control (QC) assessments on all T1w images using the IQMs pipeline from MRIQC (Esteban et al., 2017). Metrics of interest included 1) coefficient of joint variation (CJV), 2) signal-to-noise ratio for gray matter (SNR), and 3) contrast-to-noise ratios (CNR). All QC metrics fell within expected standard ranges. We also used FreeSurfer's Eueler number to evaluate a field-standard quantitative assessment of each T1w structural image. We observed no significant relationships between the Euler number and gestation week or summary brain metrics. A discrepancy (e.g., 2 SD below average) was noted in session eight; however, again, removing this session did not detract from our main findings showing reductions in gray matter volume over gestation.

T2-weighted MTL scans:
Volumes were visually assessed for QC. Volumes were removed from the analysis if unable to be reliably segmented.

Diffusion imaging:
All images were assessed using the DSI studio quality control and a visual inspection. DSI studio performed an outlier check, labeling images as a "low quality outlier" if the correlation coefficient was greater than 3 standard deviations from the absolute mean. No images were labeled as a low quality outlier.

Statistical modeling & inference

Model type and settings

Summary brain metrics:
To reflect the existing literature, we first explored brain metrics across the entire study duration (pre-conception through postpartum). When including all sessions, total brain volume, GMV, CT, global QA, ventricle volume and CSF displayed non-linear trends over time; therefore, we used generalized additive models (GAM; cubic spline basis, k = 10, smoothing = GCV), a method of non-parametric regression analysis (R package: mgcv), to explore the relationship between summary brain metrics (outcome variables) and gestation week (smooth term). Each model underwent examination (gam.check function) to ensure it was correctly specified with regards 6o 1) the choice of basis dimension (k) and 2) the distribution of the model residuals (see mgcv documentation; Wood, 2017). The general pattern of results held after toggling model parameters; however, we note the risk of overinterpreting complex models with small sample sizes (see Sullivan et al., 2015). To address overfitting and cross-validate our basis type selection, we also fit the data using nonpenalized general linear models (GLM) with both linear and polynomial terms for gestation week. We compared the performance of each GLM (i.e., models using only a linear term vs. models with polynomial terms) via the Akaike information criterion (AIC), which revealed that cubic models consistently outperformed both linear and quadratic models (AICdiff > 3), providing additional evidence for non-linear changes in structural brain variables over time.

Gray Matter Volume & Cortical Thickness:
We first computed Pearson's product-moment correlation matrices between the following variables (n = 19 pregnancy scans): gestation week, estradiol, progesterone, total GMV, and the 17 network-level average GMV values. We then ran a multivariate regression analysis predicting ROI-level GMV changes by gestation week. To identify which regions were changing at a rate different from the global decrease, we then re-ran the analyses to include total GMV as a variable of non-interest in the regression model. A similar statistical approach was taken for T1w-derived subcortical volume estimates. We ran a multivariate regression analysis predicting GMV changes over gestation in 28 regions-of-interest by gestation week (FDR-corrected at q < 0.05).

T2-weighted MTL scans:
To evaluate the relationship between gestation week and medial temporal lobe (MTL) subregion volume over pregnancy (n = 7 bilateral subregions; n = 18 MTL scans), we used a combination of linear and non-linear models based on individual subregion data patterns. Models were compared for best fit with each subregion via AIC from the GLM output (as described

above). A linear regression model was most appropriate for PHC (AICdiff < 3), whereas a quadratic model performed best for CA1 and CA2/3. As a control, we repeated the analyses with MTL subregion volumes after proportional volume correction of total gray matter volume calculated by ASHS. Finally, we evaluated the relationship between endogenous sex hormones (estrogen and progesterone) and subregion volumes using linear regression. Relationships were considered significant only if they met FDR correction at q < .05.

Diffusion imaging:
DSI Studio's correlational tractography (Yeh et al., 2016) was used to analyze the relationship between white matter structure and gestational week (n = 16). A truncated model was run to examine the relationship between white matter and sex steroid hormones (n = 14) for the subset of diffusion scans with paired endocrine data during gestation. A non-parametric Spearman correlation was used to derive the correlation between gestational week and endocrine factors and our metrics of interest (QA and MD; see Table S9 and Fig. S10 for MD results) because the data were not normally distributed. Statistical inference was reached using connectometry, a permutation-based approach that tests the strength of coherent associations found between the local connectome and our variables of interest. It provides higher reliability and replicability by correcting for multiple comparisons. This technique provides a high-resolution characterization of local axonal orientation. The correlational tractography was run with the following parameters: T-score threshold of 2.5, 4 pruning iterations, and a length threshold of 25 voxel distance. To estimate the false discovery rate (FDR), a total of 4000 randomized permutations were applied to obtain the null distribution of the track length. Reported regions were selected based on FDR cutoff (FDR < 0.2, suggested by DSI Studio), and contained at least 10 tracts. For visualization of global and tract QA at each gestational stage, mean QA values were extracted using DSI Studio's whole brain fiber tracking algorithm and ROI-based tracking using the default HCP842 atlas (Yeh et al., 2013).

Effect(s) tested: Predicting global, network, and regional volumetric change (GMV, CT, MTL subregion, microstructure) by pregnancy-related indicators (gestation week, estrogen, progesterone).

Specify type of analysis: ☐ Whole brain ☐ ROI-based ☒ Both

Anatomical location(s): Global measures of gray matter volume, cortical thickness, and cerebrospinal fluid were computed by ANTs and validated with FreeSurfer. A whole-brain probabilistic atlas (e.g., Schaefer 400-region parcellation) was used for ROI analysis of cortical thickness and volume and the Yeo/Schaefer 17-network scheme was used for network-level analyses. The 'aseg' segmentation was used for ROI analysis of subcortical gray matter volume. The Princeton Young Adult 3T ASHS Atlas Template was used to examine volume among 7 MTL subfields: CA1, CA 2/3, dentate gyrus, subiculum, entorhinal cortex, perirhinal cortex, and the parahippocampal gyrus. Whole-brain white matter structure was assessed for the diffusion imaging analysis, wherein every tract and bundle was evaluated.

Statistic type for inference: N/A; s and diffusion MRI only.

(See Eklund et al. 2016)

Correction: FDR-correction

Models & analysis

n/a	Involved in the study
☒	☐ Functional and/or effective connectivity
☒	☐ Graph analysis
☐	☒ Multivariate modeling or predictive analysis

Multivariate modeling and predictive analysis: Multivariate regression analyses was used to explore brain structure in relation to gestation. Regional, network, and summary brain measures (dependent variables) were examined in relation to gestation week (independent variable). In follow-up statistical analyses (noted in Methods), various quality control metrics and global brain volume were included into the model to account for variables of non-interest (e.g., motion) and to identify highly impacted brain areas (e.g., controlling for total GMV).
